# Synthetic Promoters and Transcription Factors for Heterologous Protein Expression in *Saccharomyces cerevisiae*

**DOI:** 10.3389/fbioe.2017.00063

**Published:** 2017-10-19

**Authors:** Fabian Machens, Salma Balazadeh, Bernd Mueller-Roeber, Katrin Messerschmidt

**Affiliations:** ^1^University of Potsdam, Cell2Fab Research Unit, Potsdam, Germany; ^2^Max-Planck Institute of Molecular Plant Physiology, Potsdam-Golm, Germany; ^3^Department Molecular Biology, University of Potsdam, Potsdam, Germany

**Keywords:** *JUB1*, synthetic biology, transcriptional regulation, gene expression, synthetic circuits, dead Cas9, chimeric transcription factors

## Abstract

Orthogonal systems for heterologous protein expression as well as for the engineering of synthetic gene regulatory circuits in hosts like *Saccharomyces cerevisiae* depend on synthetic transcription factors (synTFs) and corresponding *cis*-regulatory binding sites. We have constructed and characterized a set of synTFs based on either transcription activator-like effectors or CRISPR/Cas9, and corresponding small synthetic promoters (synPs) with minimal sequence identity to the host’s endogenous promoters. The resulting collection of functional synTF/synP pairs confers very low background expression under uninduced conditions, while expression output upon induction of the various synTFs covers a wide range and reaches induction factors of up to 400. The broad spectrum of expression strengths that is achieved will be useful for various experimental setups, e.g., the transcriptional balancing of expression levels within heterologous pathways or the construction of artificial regulatory networks. Furthermore, our analyses reveal simple rules that enable the tuning of synTF expression output, thereby allowing easy modification of a given synTF/synP pair. This will make it easier for researchers to construct tailored transcriptional control systems.

## Introduction

A major task in synthetic biology is the reconstruction, rewiring, and complete *de novo* design of transcriptional networks. Applications range from enhancing the understanding of gene regulatory and transcriptional mechanisms to reprogramming of cellular behavior, or the highly controlled expression of complete metabolic pathways (Smolke and Silver, 2011). For the latter, it is common practice to overexpress endogenous transcription factors (TFs) and/or to place the genes of interest under the control of well characterized endogenous promoters. While this is suitable for many relatively simple tasks, the complex and tunable construction of complex gene regulatory networks requires more versatile and programmable TFs and promoter components.

The use of synthetic TFs (synTFs), often based on prokaryotic TFs, enables researchers to control gene expression in many different systems (Lu et al., 2009; Weber and Fussenegger, 2009). These engineered DNA-binding proteins specifically bind to a defined target sequence, usually integrated into a hybrid promoter controlling the downstream gene of interest. A common example is the tetracycline (Tet)-dependent repressor TetR from *Escherichia coli*, which is fused to a transactivation domain (AD), e.g., the VP16 domain from the herpes simplex virus. The resulting synTF activates transcription from a corresponding Tet response element in the absence of Tet or its derivative anhydrotetracycline (Tet-off system) (Gossen and Bujard, 1992). Further examples employ the LacI or LexA proteins from *E. coli* to control heterologous gene expression (Weber and Fussenegger, 2009; Rantasalo et al., 2016). While synTFs based on bacterial repressor proteins are relatively easy to implement in transcription regulation systems, a major limitation, besides their limited number, is the generally non-variable DNA-binding specificity of these proteins. This problem persists even though the range of DNA-binding proteins can be extended, as just recently demonstrated with the use of well characterized plant TFs (Naseri et al., 2017). The missing ability to customize the DNA-binding specificity makes it virtually impossible to target endogenous promoters, to freely design novel synthetic promoters (synPs) with orthogonal binding sites (BSs), or to use the same synTF architecture to target different promoters. Furthermore, fine-tuning of such synTFs with regard to expression output is a difficult task as their binding specificity and activation potential usually cannot be modified easily. For the above reasons, synTFs with programmable DNA-binding specificity are of great interest, not only for synthetic biologists.

Customized DNA-binding proteins, based on zinc finger domains, build the foundation for the earliest type of programmable synTFs (Beerli and Barbas, 2002). By using tailored zinc finger domains to construct orthogonal synTFs for driving gene expression in yeast, Khalil and coworkers substantially contributed to the understanding and the ability to artificially design eukaryotic transcription function (Khalil et al., 2012). Furthermore, β-estradiol inducible zinc finger proteins have been successfully used to target synPs in *Saccharomyces cerevisiae* with strong gene induction 15 min post induction, thereby demonstrating their potential for the control of heterologous gene expression, e.g., in a biotechnological production scenario. However, it is a very laborious and time consuming process to generate customized zinc finger proteins with the desired target specificity (Maeder et al., 2009).

With the rise of transcription activator-like effectors (TALEs) and, more recently, RNA-guided CRISPR/Cas9-based TFs, two easy-to-handle and highly flexible classes of synTFs exist (Boch et al., 2009; Moscou and Bogdanove, 2009; Jinek et al., 2012; Wiedenheft et al., 2012). TALEs, originally derived from the plant pathogenic bacterium *Xanthomonas* spp., possess a DNA-binding domain (DBD) composed of typically 34 amino acid-long repeat units. Two amino acids within the otherwise highly conserved repeat units are hypervariable and are called “repeat variable diresidues” (RVDs) (Boch and Bonas, 2010). By following a simple code, repeat units with different RVDs can be freely combined to generate novel DNA-binding proteins with predictable and nearly unrestricted binding specificity. Target sites are usually 18–24 bp in length and start with a thymine residue (Hansen et al., 2009; Moscou and Bogdanove, 2009; Boch and Bonas, 2010; Morbitzer et al., 2011; Sanjana et al., 2012). By fusing this DBD to an effector domain, e.g., a transcriptional AD, fully functional synthetic TFs can be obtained (Zhang et al., 2011). A major drawback of TALEs is that constructing a new TALE is a relatively complex and laborious process, although it should be mentioned that the latest assembly methods allow constructing a new synthetic TALE (synTALE) within a day (Gogolok et al., 2016). However, the most recent class of synTFs, which is based on the CRISPR/Cas9 system, offers a promising alternative to synTALEs (Jinek et al., 2012; Wiedenheft et al., 2012). Hereby, a mutated version of an RNA-guided endonuclease, Cas9, from *Streptococcus pyogenes* is used. The catalytically dead Cas9 (dCas9) protein has no endonuclease activity, can be genetically fused to effector domains, and is guided to a 20-bp DNA target site via a small single-guide RNA (sgRNA) (Maeder et al., 2013; Qi et al., 2013). As the DNA-binding specificity is exclusively governed by the short sgRNA sequence, simple sgRNA cloning procedures can be applied to test large numbers of potential DNA target sites. The main limitation of dCas9-based synTFs with respect to the DNA target is the requirement of a protospacer adjacent motif (PAM, sequence “NGG”) directly adjacent to the intended BS.

The two types of synTFs described above have been successfully used to modify the expression output of target genes in various organisms, including mammalian cell lines or *S. cerevisiae* (Farzadfard et al., 2013; Maeder et al., 2013; Gao et al., 2014; Lebar and Jerala, 2016). In most cases, the synTFs are programmed to modulate endogenous gene expression by specifically binding sequences within native promoters. However, to achieve tunable and predictable expression of heterologous target genes while at the same time ensuring maximal orthogonality to the host’s endogenous gene regulation networks, synPs specifically recognized and activated by the programmable TFs and not by endogenous TFs are highly desired. To this end, synTALEs have for example been successfully developed for orthogonal regulation of gene expression in yeast and in plants, through repression and activation of corresponding (semi) synPs. (Blount et al., 2012; Brückner et al., 2015). A recent example for the successful rewiring of transcriptional functions using dCas9 is the construction of synthetic σ_70_ promoters suppressed by corresponding sgRNAs. These were used to construct multi-layered genetic circuits, interconnected with the *E. coli* transcriptional network (Nielsen and Voigt, 2014). Cress et al. (2016) generated a panel of modified T7 promoters that can be specifically and orthogonally repressed by corresponding sgRNAs. A set of these synPs was used to drive the heterologous expression of the five-gene violacein biosynthesis pathway in *E. coli*. An impressive example for complex transcriptional circuits in eukaryotic cells involves the use of a dCas9-Mxi1 repressor protein in yeast that was employed to generate multi-layered genetic circuits with a high degree of orthogonality and digital responses (Gander et al., 2017).

In the study presented here, we constructed and characterized a collection of synTFs, based on dCas9 and synTALEs, along with their corresponding synPs. The library is especially designed for heterologous gene expression in the yeast *S. cerevisiae*. We focused on providing a versatile, ready-to-use collection of different synTF/synP pairs that exhibit minimal background activation, cover a wide range of low to high expression outputs upon synTF induction, and can be easily fine-tuned according to the experimenter’s needs.

## Materials and Methods

### Plasmid Construction and Yeast Transformation

Plasmids pLOG1 and pUOGB were gifts from Tom Ellis, Imperial College London (Ellis et al., 2009). Plasmid p426-SNR52p-gRNA.CAN1.Y-SUP4t was a gift from George Church (Addgene plasmid # 43803) (DiCarlo et al., 2013). Plasmid pMLM3705 was a gift from Keith Joung (Addgene plasmid # 47754) (Maeder et al., 2013). The plasmid kit used for building TALE-TFs was a gift from Feng Zhang (Addgene kit # 1000000019) (Zhang et al., 2011; Sanjana et al., 2012). All DNA manipulations were done according to standard procedures, using restriction—ligation, Gibson assembly, SLiCE cloning or *in vivo* recombination in yeast. Plasmid maps and nucleotide sequences of plasmids generated in this work are shown in Figures S2–S4 and Data File S1 in Supplementary Material. Yeast transformations were done with the LiAC/PEG method (Gietz and Schiestl, 2007), using YPH500 cells (ATCC^®^ 76626™). To create expression plasmids for synTALE1–10, plasmid pLOGI was modified: for construction of plasmid pFM003B, the *Age*I restriction site was deleted, the *Stu*I site was replaced by *Pme*I and the yEGFP CDS was replaced by the SV40NLS (PKKKRKV) and the GAL4-AD (both amplified from pGAD424, Takara Bio, Saint-Germain-en-Laye, France) with *Bam*HI and *Age*I restriction sites in between. For plasmid pFM004B, the *Bam*HI and *Age*I sites are positioned at the C-terminal site of the SV40NLS-GAL4-AD. Reporter plasmid pFM005 was created by insertion of the *CYC1* minimal promoter with upstream *Sal*I and *Xba*I sites into *Bam*HI/*Eco*RI digested pUOGB. Control plasmid pFM006 was constructed by assembling *Bam*HI/*Eco*RI digested pUOGB with the *TDH3* promoter, which was amplified from the YPH500 genome. To create the dCas9 expression plasmid, the *Stu*I site in pLOGI was replaced by *Pme*I and the yEGFP CDS by the dCas9-VP64 fusion from pMLM3705. The plasmid p426-SNR52p-gRNA.CAN1.Y-SUP4t was modified by introducing a *Bam*HI-*Eco*RI cloning site between the *SNR52* promoter and the structural sgRNA backbone and by exchanging the *URA3* marker for the *LEU2* marker gene, resulting in pFM021. Digestion of pFM021 with *Bam*HI/*Eco*RI allows overlap-based cloning of double-stranded oligonucleotides containing the desired sgRNA sequence. SynTALE cloning vectors pTALE-TF_v2_NG, pTALE-TF_v2_NN, pTALE-TF_v2_HD, and pTALE-TF_v2_NI from the TALE kit were modified by amplifying the TALE backbones flanked with *Bam*HI and *Age*I sites and subsequent cloning into pFM0011 to give plasmids pFM0027_HD, pFM0028_NG, pFM0029_NI, and pFM0030_NN. To allow yeast-based expression of synTALE11–15, plasmid pFM031 was created: the SV40NLS-GAL4-AD fusion from pFM004B was replaced by *Bam*HI and *Age*I sites upstream of an SV40NLS-VP64-AD fusion. Plasmid pFM033 was created by deletion of both *Bsm*BI sites from pUC19, digestion of the resulting plasmid with *Hin*dIII and *Eco*RI and assembly with a double-stranded oligonucleotide containing the following restriction sites: *Sal*I, *Hin*dIII, *Bam*HI, and *Age*I.

### Construction and Subcloning of synTALEs

The five different synTALEs targeting the *JUB1* promoter sequences were cloned using the TALE repeat assembly kit as described in Morbitzer et al. (2011). To this end, three distinct level 2 modules that contain 5, 5, and 10 repeat modules (TALE RVD monomers), respectively, were assembled. Complete synTALEs were then digested with *Bam*HI/*Age*I to release functional DBDs. The fragments were subcloned into *Bam*HI/*Age*I digested pFM003B and pFM004B to give functional synTFs with C- or N-terminal GAL4-AD, respectively. Resulting plasmids were linearized with *Pme*I, integrated into the yeast YPH500 genome at the *ura3-52* locus and selected on SD-Trp medium. Similarly, the second set of synTALEs, constructed in the modified cloning vectors pFM00027–pFM00030 by using the TALE toolbox (Zhang et al., 2011; Sanjana et al., 2012), was subcloned into pFM031 via *Bam*HI/*Age*I to give synTALEs with C-terminal SV40NLS and VP64-AD, and subsequently integrated in the YPH500 genome.

### Construction of synPs for synTFs

Binding sites for synTALE1–10 were cloned into pEGFP_Y1H, a derivative of pHis2.1 (Takara Bio), in which the *HIS3* reporter gene was exchanged for *yEGFP*. BSs were non-directionally cloned into the *Mlu*I restriction site as annealed oligonucleotides (Table S4 in Supplementary Material). Each pair of oligonucleotides contains four identical BSs, separated by *Nhe*I sites. Clones harboring a forward or reverse version of the BS array were selected. *Nhe*I digestion and recircularization resulted in clones containing two copies of each BS. *Nhe*I/*Spe*I digestion and recircularization resulted in a single BS. All BSs were subcloned into *Xba*I/*Sal*I digested pFM005. BSs for synTALE11–15 and dCas9-based TFs were cloned following a different strategy, to allow higher copy numbers: pFM033 was digested with *Bam*HI and *Hin*dIII and ligated with double-stranded oligonucleotides (Table S4 in Supplementary Material). The oligonucleotides contain two copies of the BS. Upstream and downstream of the target site array a *Btg*ZI and a *Bsm*BI site, respectively, and appropriate linker sequences are present. Positive clones were cut with *Aat*II and *Bsm*BI, and *Aat*II and *Btg*ZI in two separate reactions. The large fragment from the *Aat*II/*Bsm*BI digestion was ligated with the small fragment from the *Aat*II/*Btg*ZI reaction. Resulting plasmids contain four copies of the BS. Repetition of the procedure leads to doubled BS copy number with each iteration. Target site arrays were subcloned into pFM005. Reporter vectors were linearized with *Aat*II and transformed to the yeast strains containing the expression cassette for the corresponding synTF. Integration into the yeast genome takes place within the already integrated pFM003B or pFM004B backbone. Clones were selected on SD-Trp/-His medium. The integrated synPs were size-verified by PCR amplification to exclude rearrangements due to the repetitive targets sequences and sequenced on a sample basis. If not stated otherwise, three independent clones were used for the subsequent analysis of synTF-mediated reporter gene activation.

### Induction of synTFs and Measurement of yEGFP Fluorescence

To measure synTF-mediated yEGFP fluorescence, yeast reporter strains were used to inoculate 500 µL SD-Trp/-His medium in 48-well deepwell plates (or SD-Trp-His-Leu for dCas9 + sgRNA experiments). Plates were incubated with shaking for 20–24 h at 30°C and 230 rpm. The main culture in YPDA and YPA-Gal (galactose: 20 g/L) + 20 mM IPTG was inoculated to an OD_600 nm_ ≈ 0.1 in three technical replicates. The culture was grown at 30°C at 230 rpm for 14–16 h. Cycloheximide was added to a final concentration of 500 µg/mL and samples were subsequently analyzed using a BD FACSCalibur flow cytometer with a 488 nm excitation laser and 530/30 nm band pass filter for detection (BD Biosciences, Heidelberg, Germany). *S. cerevisiae* cells were diluted in water and passed through the cytometer with less than 3,000 counts per second. A total of 20,000 cells were counted per measurement. The geometric mean fluorescence per cell was calculated, using Flowing Software 2 (version 2.5.1, www.flowingsoftware.com). If not stated otherwise, results shown are mean values derived from the three independent yeast transformants with three technical replicates each. Error bars indicate the standard deviation. For the time course experiment, precultures were grown as described above. For the main culture, 150 µL of fresh inducing or non-inducing medium were inoculated with 5 µL preculture in a transparent 96-well cell culture plate (Thermo Fisher Scientific Inc., Cat. No. 167008). To prevent evaporation, the culture was overlaid with 50 µL mineral oil. Measurement of yEGFP fluorescence and OD_600 nm_ over a 48-h period was done in a TECAN Infinite M200 Pro plate reader with monochromator optics (TECAN Group Ltd., Maennedorf, Switzerland) at 30°C with the following continuous measurement cycle: 12 shaking cycles, each with 60 s linear shaking (6 mm amplitude) and 60 s orbital shaking (6 mm amplitude), directly followed by fluorescence (excitation wavelength: 475 nm, emission wavelength: 516 nm) and OD_600 nm_ readings. This was repeated for 48 h. OD and fluorescence readings were corrected by the corresponding blank value (medium without cells for OD readings and non-fluorescent wild-type YPH500 cells for fluorescence measurements). Each synTF was measured from two independent colonies, with two technical replicates each.

## Results and Discussion

Synthetic promoters and corresponding synTFs can be used to regulate the expression of heterologous genes without extensively relying on endogenous host TFs. A tunable and reasonable range of expression strengths is desired, especially when novel biosynthetic pathways are to be implemented. Here, we investigate the use of TALE- and dCas9-based synTFs together with small-size synPs to drive gene expression in yeast. To this end, we developed a set of reporter and expression plasmids, based on a previously published system (Khalil et al., 2012). These plasmids allow the sequential genome integration of an isopropyl β-d-1-thiogalactopyranoside (IPTG)-controlled synTF expression cassette and a corresponding synP-yEGFP reporter construct. synTF expression is controlled by *LX*, a derivative of the yeast *GAL1* promoter, containing the *E. coli lac* operator (*lacO*) site. In the absence of IPTG, the *lacO* site is bound by the LacI repressor, which is constitutively expressed from the *TEF1* promoter (Ellis et al., 2009). Upon IPTG induction, the repressor is released from the operator, the synTF is expressed, and yEGFP fluorescence is used as a measure for synTF-controlled gene expression.

Synthetic TALEs were assembled by two different strategies. A first set of five synTALEs, targeting 19-bp long sites (including the mandatory thymine residue in position 0) of the *Arabidopsis thaliana JUB1* promoter (AGI: At2g43000), were assembled according to Morbitzer et al. (2011) and subcloned into the genome-integrating yeast plasmids pFM003B and pFM004B. The subcloned fragments encode the full N-terminal region of the synTALEs, the complete DBD (established on NK RVD frameworks), and the C-terminal domain, shortened by the last 89 amino acids. Depending on the expression vector used, the synTALE was fused either to the N- or the C-terminus of the GAL4-AD. The resulting synTFs were designated synTALE1 to synTALE10 (Table 1). The corresponding 19-bp target sites were analyzed for occurrence in the *S. cerevisiae* genome. For BS1 to BS4, a BLAST analysis revealed a maximum of 14 consecutive nucleotides identical to regions in the yeast SC288c genome. As synTALEs with 18 RVDs tolerate no more than 1–2 bp mismatches in their target region, these sequences are most likely not bound by the synTALEs used here (Mali et al., 2013). A complete 19-bp hit was found for BS5 in the bidirectional terminator of the genes *PET54* and *HSV2*. Nevertheless, we employed the corresponding synTALE in our study, as the location of its BS within a terminator region makes it unlikely to severely affect the host’s gene expression or to represent an endogenous *cis*-regulatory motif.

**Table 1:** Synthetic transcription factors (synTFs) and target sites used in this study.

synTF	Activation domain (AD) type	Binding site (BS)	BS sequence^a^
synTALE1	C-terminal GAL4-AD	BS1	TCTATAAGATCTTGTGTGC
synTALE2	N-terminal GAL4-AD		

synTALE3	C-terminal GAL4-AD	BS2	TAGTCAAAGTCATTCGTAA
synTALE4	N-terminal GAL4-AD		

synTALE5	C-terminal GAL4-AD	BS3	TGACCAAGCACCAATTAAA
synTALE6	N-terminal GAL4-AD		

synTALE7	C-terminal GAL4-AD	BS4	TAATCAATAAATAGATAAA
synTALE8	N-terminal GAL4-AD		

synTALE9	C-terminal GAL4-AD	BS5	TATATATATGTATAGAGAA
synTALE10	N-terminal GAL4-AD		

synTALE11	C-terminal VP64-AD	BS11	TGAAATGCTGACCATGAATT

synTALE12	C-terminal VP64-AD	BS12	TAGACGATAGCTCAGGGAGA

synTALE13	C-terminal VP64-AD	BS13	TGTTCTCGAACGGAGAGATA

synTALE14	C-terminal VP64-AD	BS14	TCCTCTCTGTCGTCGCTAAC

synTALE15	C-terminal VP64-AD	BS15	TTGTAAGTACTTAATCTCAT

dCAS9	C-terminal VP64-AD	Determined by single-guide RNA	

*^a^Underlined nucleotides are directly targeted by the transcription activator-like effector (TALE) repeat variable diresidue, while the thymine residue at position 0 is mandatory for TALE binding*.

A second set of five synTALEs was created using a method reported by Sanjana and colleagues (Sanjana et al., 2012). As opposed to synTALE1–10, these use NN RVDs rather than NK RVDs to target guanine nucleotides. NN-type TALE nucleases (TALENs) have been reported to exhibit higher activity and higher specificity than NK-type TALENs (Christian et al., 2012; Streubel et al., 2012). A similar behavior may be expected for NN-type synTALE-based TFs. The DBDs were designed to target five *de novo* designed 20-bp sequences with a GC content of 35% and a maximum of 14 consecutive nucleotides identical to the *S. cerevisiae* genome (Table 1). The GC content was set to 35% to reflect the average GC content in yeast promoter regions (Erb and van Nimwegen, 2011). Cloning of these synTALEs into the integrating yeast vector pFM031 resulted in synTALE11–15, each fused to a C-terminal VP64-AD (composed of four copies of the viral VP16-AD).

Similar to the synTALE expression system, we placed a dCAS9-VP64-AD fusion protein under control of *LX* on the integrative plasmid pLOGI (Maeder et al., 2013). The *SNR52* promoter–driven sgRNA expression cassettes, necessary to direct the dCAS9-VP64-AD proteins to their DNA targets, were designed to target the same 20-bp sequences as synTALE11–15.

**Figure 1:**
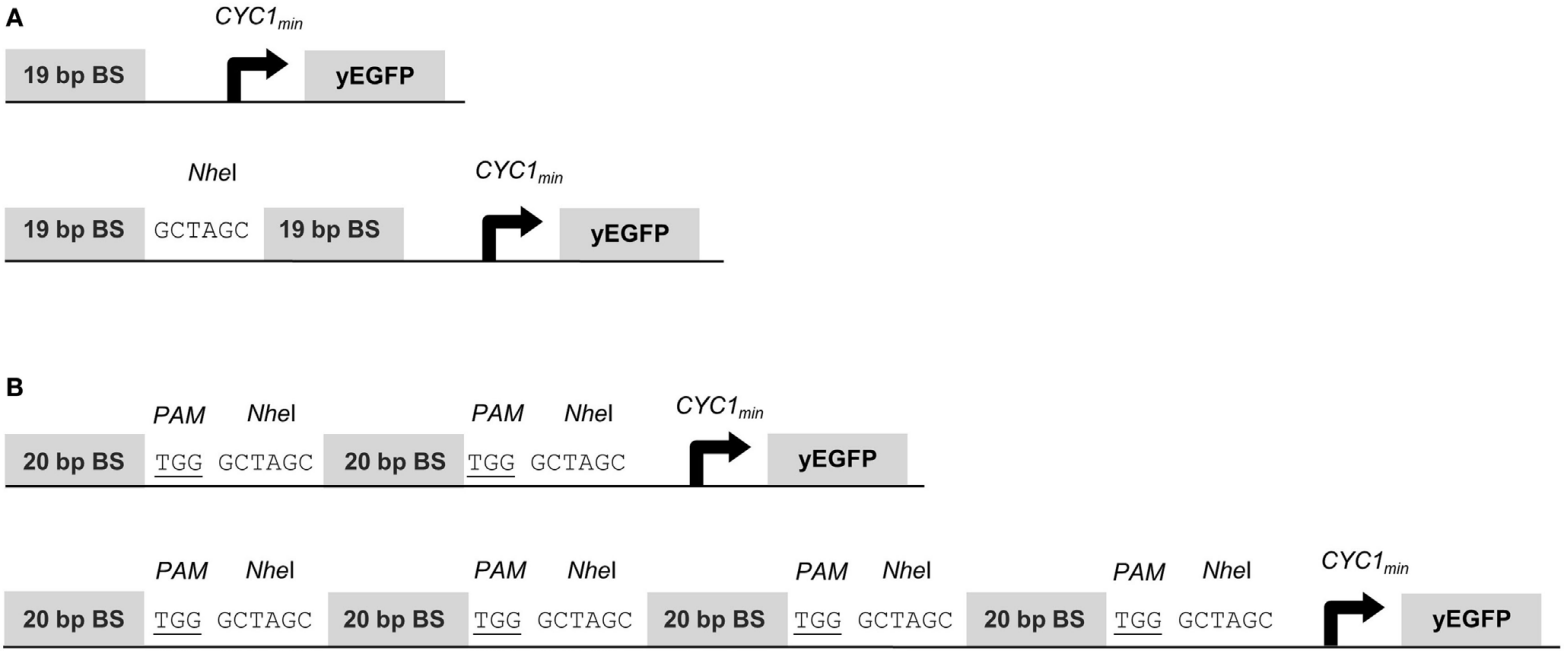
Structure of synthetic promoters. The *CYC1* minimal promoter (*CYC1_min_*) is located downstream of the binding site (BS) array and controls yEGFP expression. **(A)** BSs for synthetic transcription activator-like effectors (synTALEs) 1–10 are 19 bp long. Up to four direct repeats of the same BS are separated by *Nhe*I sites. **(B)** BSs for synTALE11–15 and dead Cas9 (dCas9)-based transcription factors (TFs) are 20 bp long. Direct repeats are flanked by the PAM and a *Nhe*I site.

To assess gene activation potentials of the synTFs described above, we placed the yEGFP reporter gene under the control of the *CYC1* minimal promoter (*CYC1_min_*) and a variable copy number of upstream BSs for the synTF under investigation. The 19-bp BSs for synTALE1–10 were cloned as monomers, dimers and tetramers, with a 6-bp spacer sequence between the individual repeats (Figure 1A). Each BS array was inserted in forward and reverse orientation. Equally, the 20-bp target sites for synTALE11–15 and the dCas9-based synTF were inserted upstream of *CYC1_min_*. Here, the copy number was varied between 2 and 16. To achieve this, we employed a simple cloning strategy that enables the rapid multimerization of BSs in a synP and allows the construction of large BS arrays, even beyond 16 BS copies (see Materials and Methods for details). Here, the BSs were used in forward orientation only and were separated by a *Nhe*I site and the PAM sequence “TGG” to allow sgRNA-mediated dCAS9 binding. The resulting spacer sequence between the BSs is 9 bp in length (Figure 1B).

**Figure 2:**
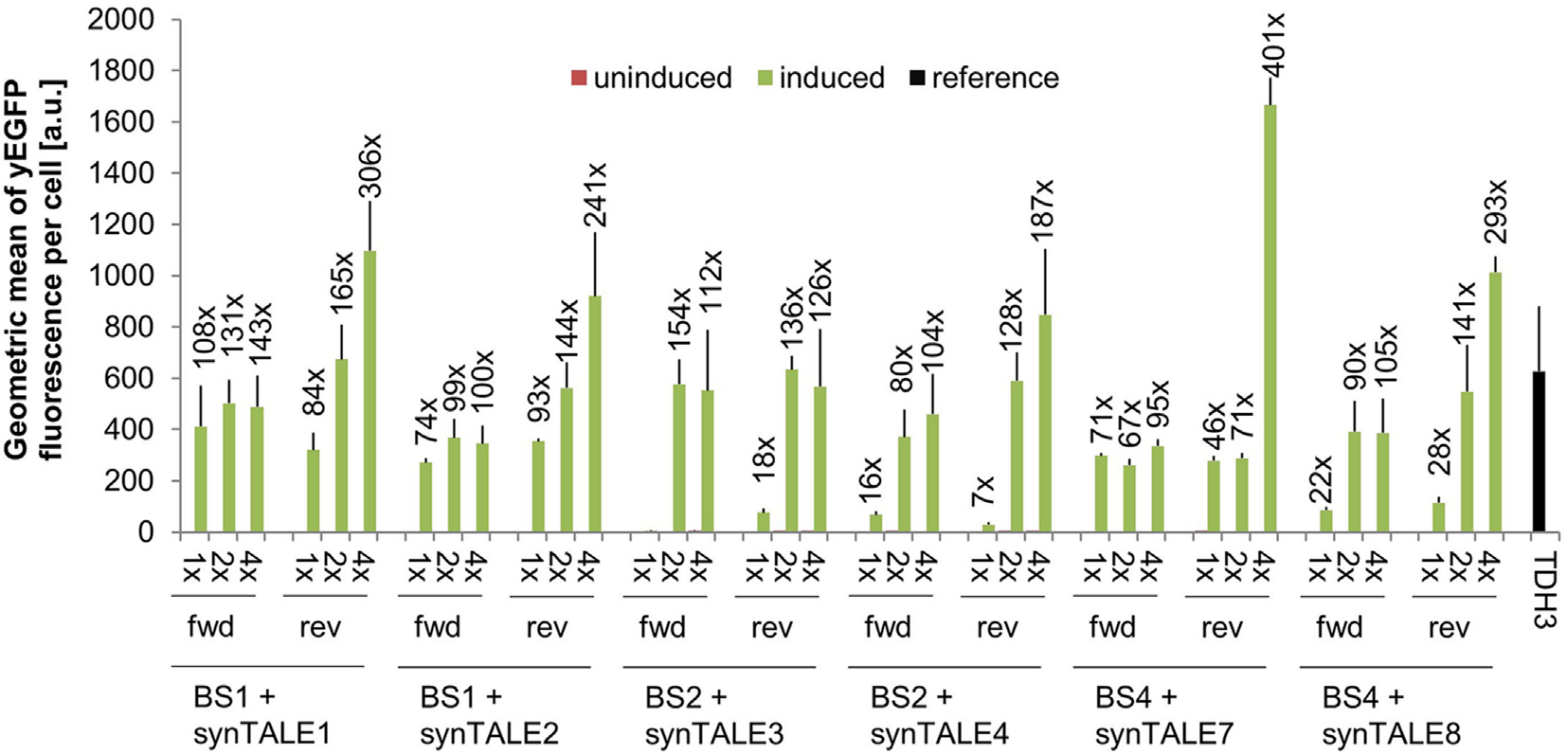
Synthetic transcription activator-like effectors (synTALEs) confer inducible reporter gene expression from synthetic promoters (synPs). yEGFP fluorescence of yeast cells with corresponding synTALE/synP pairs was analyzed by flow cytometry. SynTALE expression was induced with IPTG in galactose containing medium. Background activity of synPs was measured in glucose containing medium without IPTG. Values from uninduced samples are always very low. Induction of at least 2-fold is indicated. 1×, 2×, 4×: binding site (BS) copy number. Fwd, rev, forward/reverse orientation; a.u., arbitrary units; TDH3, *TDH3-yEGFP* measured in medium without IPTG.

The combination of synTALE1–10 with all versions of the corresponding synPs resulted in a set of 60 different synTF/synP pairs. Flow cytometry-based analysis of yEGFP fluorescence revealed very low basal activity under non-inducing conditions for all synTALEs. The basal level of fluorescence is—with a minor exception for synTALE6—nearly identical to that of yEGFP expression driven by the *CYC1_min_* promoter without additional upstream BSs (Table S1 in Supplementary Material). This indicates that the BSs incorporated upstream of the *CYC1_min_* promoter are indeed orthogonal to yeast and do not act as endogenous *cis*-activating sequences, as the synPs are obviously not activated in the absence of the corresponding synTF. This is an important feature for applications that require tight expression regulation, e.g., for the expression of toxic proteins. Upon induction of synTALE expression, we observed no or only very weak induction in the case of synTALE5, 6, 9, and 10 (Table S1 in Supplementary Material). SynTALE5 and synTALE6 are based on the same DBD, targeting sequence BS3. Equally, synTALE9 and synTALE10 are derived from a common DBD, designed to bind BS5. The lack of gene activation suggests inefficient binding of both DBDs targeting BS3 and BS5, regardless of the AD position within the fusion protein. The remaining six synTALEs, based on three different DBDs, confer higher fluorescence output. Induction factors compared to non-induced samples range from as low as 1.4- to 400-fold (Figure 2). In general, we observed a positive correlation between the number of BS copies in a given synP and the fluorescence output: In most cases, increasing the number of target sites from a single copy to two copies results in a higher fluorescence. This suggests an additive effect of multiple synTALEs binding the same synP, which has been described before and enables the easy tuning of the promoter output (Khalil et al., 2012; Perez-Pinera et al., 2013). However, when the BS copy number is further increased to four, this effect is mainly observed for BSs placed in reverse orientation. This results in overall better performance of synPs with multiple reverse BSs. As opposed to the BS orientation, changing the position of the GAL4-AD within the synTF has mostly no or only minor effects. A possible reason for the better performance of synPs with reverse oriented BSs in the experiment described here may be steric hindrance that disturbs the interaction of the GAL4-AD with the basal transcription machinery. However, such effects may also be expected for synTFs harboring the GAL4-AD in different positions. Remarkably, the expression output of the strongest synTALE/synP pairs (e.g., synTALE1 with 4× BS1) is even higher than the fluorescence of yEGFP expressed from the yeast *TDH3* promoter (Figure 2; Table S1 in Supplementary Material). The *TDH3* promoter has been described as one of the strongest constitutive yeast promoters and is commonly used for high-level expression of heterologous genes in yeast (Mumberg et al., 1995; Sun et al., 2012; Peng et al., 2015). Thus, our synTALE/synP pairs may be very useful for applications requiring high levels of inducible gene expression with minimal background activity.

For tuning the transcription output of a synP, altering the BS copy number seems to be a good starting point, in particular for reverse-oriented BSs. Changing the position of the GAL4-AD within the synTALE, however, does not lead to a predictable outcome and is thus not a good option for directed modulation of target gene expression.

**Figure 3:**
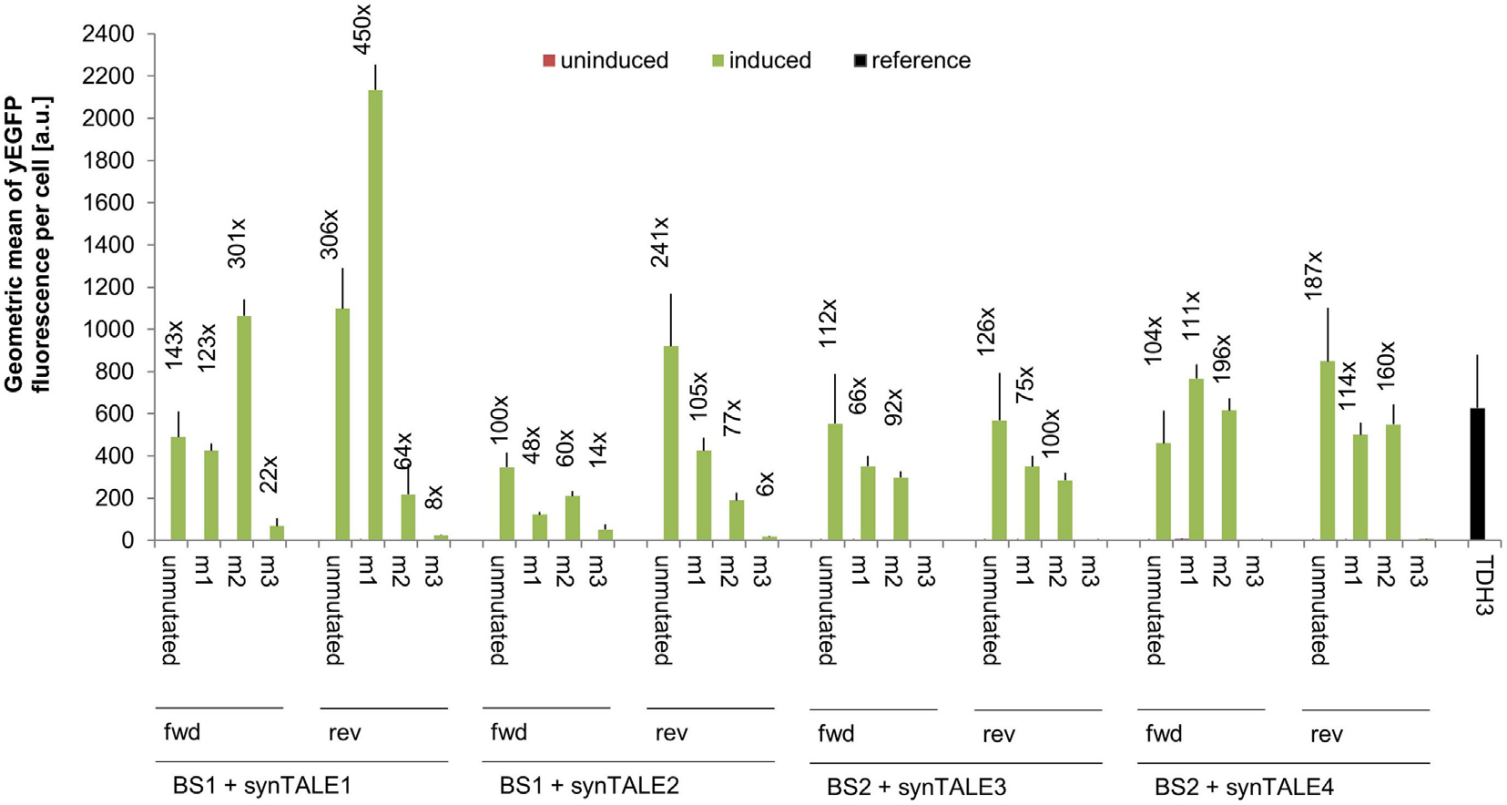
Mismatches in synthetic transcription activator-like effector (synTALE)-binding sites (BSs) in most cases gradually reduce the transactivation capacity. SynTALE1–4 were tested for transactivation of yEGFP expression from synthetic promoters (synPs) containing mutated versions (m1, m2, and m3) of their cognate BS. SynTALE expression was induced with IPTG in galactose containing medium. Background activity of synPs was measured in glucose containing medium without IPTG. Values from uninduced samples are always very low. Induction of at least 2-fold is indicated. Fwd, forward orientation; rev, reverse orientation; a.u., arbitrary units.

We next tested whether fine-tuning the transcriptional output is possible by reducing the binding affinity of synTALEs to their cognate BSs; this was achieved by mutating one to three nucleotides within BS1 and BS2. All point mutations are guanine-to-adenine exchanges, thereby creating mismatches to the guanine-specific RVD NK, used in the corresponding synTALE1–4 (Table 2). By introducing mismatches to the weak NK RVD, we intended to cause a moderate, stepwise reduction in overall binding affinity. Each synTALE was tested for its activation potential using synPs containing the mutated BS in four tandem copies, either in forward or reverse orientation. Generally, one and two mismatches within the BS are well tolerated by all four synTALEs tested (Figure 3), although, as expected, the yEGFP fluorescence upon induction is mostly lower than for the original BS. However, substantial gene activation potential remains, leading in some cases to even higher transcriptional output than with the perfect BS. This observation indicates that even in the presence of a relatively robust RVD code, other parameters affect the efficiency of the synTF–synP interaction or the transcription activation capacity of the synTALEs, supporting earlier conclusions that, e.g., the pure RVD code is not sufficient for predicting BS affinity (Meckler et al., 2013; Juillerat et al., 2014; Lin et al., 2014). As expected, the introduction of a third mismatch results in a nearly complete loss of inducible yEGFP fluorescence, indicating a strongly reduced binding affinity of the synTALE1/2 and 3/4 to BS1_m3 and BS2_m3, respectively. Our results demonstrate that introducing single nucleotide mismatches into the BS can be sufficient for fine-tuning synTALE-mediated transcription control, whereby a moderate change in expression strength is achievable by introducing 1–2 mismatches. We expect that in most cases it will be sufficient to randomly generate mutated versions of a given BS to establish a desired expression output. Thus, this approach to generate new synTF/synP combinations with slightly modulated expression output is an interesting alternative to the relatively time consuming *de novo* assembly of a new synTALE with an altered DBD.

**Table 2:** Mutated binding sites (BSs).

BS	BS sequence
**BS1**	TCTATAAGATCTTGTGTGC
**BS1_m1**	TCTATAAAATCTTGTGTGC
**BS1_m2**	TCTATAAAATCTTATGTGC
**BS1_m3**	TCTATAAAATCTTATGTAC
**BS2**	TAGTCAAAGTCATTCGTAA
**BS2_m1**	TAATCAAAGTCATTCGTAA
**BS2_m2**	TAATCAAAATCATTCGTAA
**BS2_m3**	TAATCAAAATCATTCATAA

*Exchanged nucleotides are underlined*.

**Figure 4:**
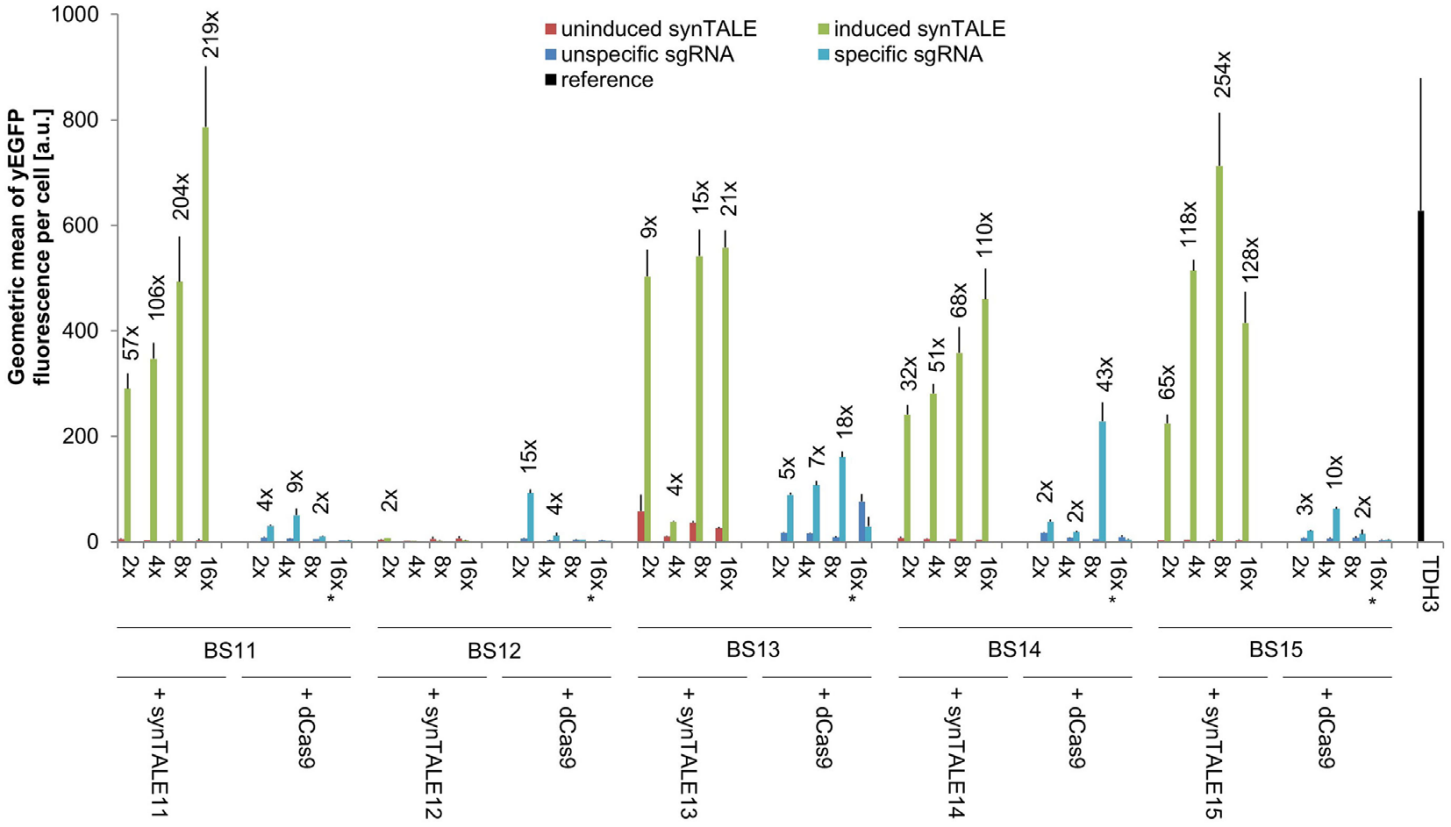
Transactivation potential of synthetic transcription activator-like effectors (synTALEs) 11–15 and dCAS-VP64. synTALE11–15 and dead Cas9 (dCas9)-VP64 were tested for their potential to activate yEGFP expression from different synthetic promoters (synPs), each harboring 2, 4, 8, or 16 copies of the corresponding binding site (BS). SynTALE expression was induced with IPTG in galactose containing medium. Background activity of synPs was measured in glucose containing medium without IPTG. dCas9 experiments were done in galactose containing medium with IPTG to promote dCas9 expression. yEGFP fluorescence was measured in the presence of either an single-guide RNA (sgRNA) specific for the synP or an unspecific control sgRNA. Values from uninduced samples are very low. Induction of at least 2-fold is indicated. 2×, 4×, 8×, and 16× indicate the BS copy number. Fwd, forward orientation; rev, reverse orientation; a.u., arbitrary units. Values are mean values from 3 or 2 (indicated with *) independent experiments.

In the next step, we analyzed the activation potential of synTALE11–15 in comparison with dCas9-based TFs, targeting the same BSs. synTALE11–15 and dCas9 are both fused to the VP64-AD. The results for synTALE11–14 are similar to our findings for synTALE1–10: the basal activity of the synPs is again very low, regardless of the number of BSs incorporated in a specific synP. The only exceptions are synPs containing BS13, for which slightly higher background fluorescence is detectable. Upon induction of synTF expression, we observed strong yEGFP fluorescence for synTALE11, 13, 14 and 15. Fluorescence levels of the strongest synTFs are similar to the previously mentioned strong *TDH3* promoter. However, the maximum expression is lower than for synTALE1–10 (Figure 4). Only synTALE12 does not confer any yEGFP fluorescence. The reason for the lower transactivation potential of synTALE11–15 compared to synTALE1–10 may be the exchange of the GAL4-AD by the VP64-AD, suggesting a stronger activation potential by the endogenous yeast AD and/or a lower target affinity of the NN RVD containing synTALE11–15. The latter would be surprising as NK RVDs, used in synTALE1–10, were reported to be weaker than NN RVDs (Christian et al., 2012; Streubel et al., 2012). Increasing the number of BSs in the synPs generally increases the output. Compared to synTALEs with a GAL4-AD the additive effect observed in synTALE-VP64-AD fusions is more pronounced and appears to be less variable. This makes it easier to predict the outcome of an increased BS copy number. Assuming that this effect is due to the different ADs, the use of the VP64-AD may be favorable for easy promoter fine-tuning. Regarding the modulation of transcriptional output, it may also be interesting to evaluate the effect of variable spacer length between individual BS copies within a synP. In this study, we used a 6- and 9-bp spacer for synTALE1–10 and synTALE11–15, respectively. The more pronounced additive effect of additional BS copies for synTALE11–15 may also be due to the slightly longer spacer sequence used for these synTFs. Spacing between individual BSs has been reported to affect transcriptional activity, possibly due to altered binding dynamics of multiple TFs binding in close proximity to each other (Murphy et al., 2007). In conjunction with the simple and effective BS multimerization strategy we employed here, spacer length is yet another variable that can be addressed for promoter fine-tuning.

A direct comparison of synTALE11–15 with dCas9-based TFs, targeted to the same BSs, reveals weaker and less robust gene activation through dCas9 (Figure 4; Table S2 in Supplementary Material). With the exception of BS12, the maximum fluorescence output for the BSs tested is considerably lower than for the synTALE. In most cases, the yEGFP fluorescence for dCas9-based activation reaches roughly 10% of the corresponding synTALE-mediated expression. The weaker transactivation capacity of dCas9-based TFs compared to synTALEs targeting the same BS has just recently been reported (Lebar and Jerala, 2016). Interestingly, increasing the copy number of BSs for dCas9 has variable effects. For BS13, a clear elevation of yEGFP fluorescence can be observed upon increasing the copy number from two to four, or eight copies. This is consistent with previous observations (Maeder et al., 2013; Lebar and Jerala, 2016). However, a sharp decrease occurs for 16 BSs. Similarly, for BS12, BS14, and BS15 a clear optimum for the BS copy number can be observed which varies between two and eight copies. It is likely that this difference to synTALE11–15, where more BSs usually result in higher activity, is related to the dCas9-specific mode of DNA-binding. dCas9 requires a single-stranded DNA section to allow base pairing between the sgRNA and the BS. This may have negative effects when too many BSs exist in near proximity to each other. The dCas9-mediated gene activation might therefore be positively influenced by increasing the distance between the individual BSs in a synP. Based on the results presented here, it can be concluded that TALE-based synTFs represent the more robust type of transcriptional activator, requiring less optimization of corresponding synPs in order to achieve high expression output.

**Figure 5:**
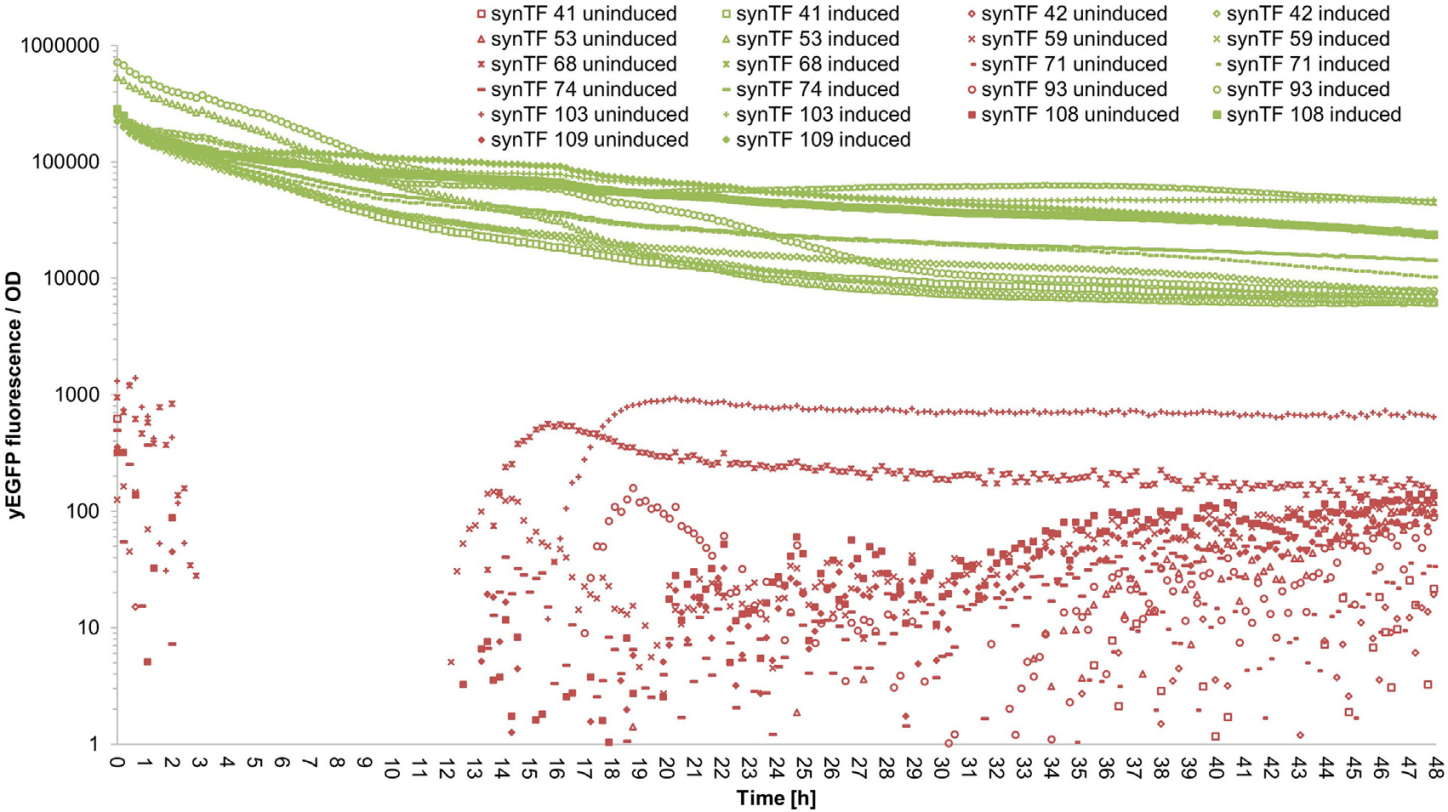
Fluorescence/OD values of selected synthetic transcription factors (synTFs) over a 48-h cultivation period. Cells expressing synTFs were grown in inducing and non-inducing medium in a 96-well plate. Fluorescence and OD_600 nm_ readings were taken approximately every 12 min. Fluorescence values were divided by the corresponding OD to account for differences in cell density. synTF numbering refers to Table S3 in Supplementary Material. Values are mean values from two biologically independent replicates, each with two technical replicates. For better readability, standard deviations are not indicated here, but given in Table S5 in Supplementary Material.

To evaluate if our synTF/synP pairs confer robust and continuous transcriptional activation over the complete course of a typical cultivation period, we performed a time course experiment: We selected 11 synTALE/synP pairs (# 41, 42, 53, 59, 68, 71, 74, 93, 103, 108, and 109, numbers according to Table S3 in Supplementary Material) from our collection, thereby covering both synTALE architectures (NN and NK types) and a range of different expression strengths. For the selected synTF/synP pairs, we measured the whole-culture fluorescence intensities and the OD_600 nm_ of induced and non-induced samples over 48 hours in a microplate reader. To account for different cell densities and growth rates, the fluorescence intensity was divided by the respective OD_600 nm_ at each time point. As compared to non-induced samples, the OD-corrected fluorescence values for the selected synTFs demonstrate a robust reporter gene activation over the complete 48 h period. However, during the first 10–20 h of cultivation the fluorescence/OD values constantly decline before a stable plateau is reached (Figure 5 for an overview and Figures S5–S15 in Supplementary Material for detailed graphics). For most of the synTFs tested, inspection of separate OD and fluorescence values reveals that during this period both variables increase, with the OD_600 nm_ increasing at higher rates, resulting in the observed declining ratio. Of note, the performance of the synTFs during this experiment is most likely mainly a function of the *LX* promoter driving synTF expression. According to the experimental needs, this promoter can be exchanged to any other promoter. However, the results for the *LX* driven synTFs demonstrate their general robustness over longer incubation periods.

In the context of the time course experiment, it should be mentioned that the yeast strains harboring the 11 synTFs tested exhibit near wild-type growth under non-inducing conditions, while they show reduced growth rates upon induction (Figures S5–S15 in Supplementary Material). Given the change of the sugar source in the induction medium (glucose to galactose) and the metabolic burden through high-level transgene expression, this behavior is not unexpected but should be kept in mind when high cell densities are required for a specific experiment.

Taken together, the synTF/synP pairs analyzed here cover a wide range of inducible expression strengths (Figure S1 and Table S3 in Supplementary Material), rely only on a minimum of endogenous yeast components, and confer nearly no detectable expression in the absence of their cognate synTF. This makes them especially useful for the construction of orthogonally regulated signaling or biochemical pathways. For example, simple genetic circuits may be constructed by placing one or more synTFs under control of a synP, which is targeted by a master regulator, e.g., a synTF driven by the IPTG-inducible *LX* promoter. The synTFs regulated by the master regulator can then be used to drive expression of synP-controlled target genes, e.g., biosynthetic genes. In this way, a single input signal (e.g., IPTG) can be used to simultaneously control target genes within multi-gene pathways, while allowing individually selected expression levels. For such purposes, the synTF/snyP pairs can be integrated into existing cloning frameworks for synthetic biology, for example, the AssemblX toolkit, which allows simple, software-assisted multi-gene assemblies for different host organisms (Hochrein et al., 2017a). This was successfully demonstrated in a recent study, where one of the synTALEs described here was integrated into a light-dependent gene activation system (PhiReX). In this system, the synTF was constitutively expressed and conferred red light inducible and far-red light reversible gene activation over at least 48 h (Hochrein et al., 2017b). The particular small size of the synPs used in this study and our simple but effective BS multimerization strategy ensure easy handling and modification. The weaker and less reliable activation through dCas9-based synTFs renders the synTALEs the favorable solution for most applications. However, the easier to handle and more flexible dCas9-based synTF/synP pairs characterized here can still be useful for experiments that do not require highest expression output.

## Author Contributions

FM designed and conducted the experiments, analyzed the data, and wrote the paper. SB designed and cloned the synTALEs targeting BS1–5. BM-R designed the overall research strategy and edited the paper. KM supervised the Cell2Fab lab and proofread the paper.

## Conflict of Interest Statement

The authors declare that the research was conducted in the absence of any commercial or financial relationships that could be construed as a potential conflict of interest.

## Supplementary Material

Data Sheet S1 GenBank sequences for plasmids generated in this work.
