# Supplementary material for: Synthetic Promoters and Transcription Factors for Heterologous Protein Expression in Saccharomyces cerevisiae
Source: Front Bioeng Biotechnol. 2017 Oct 19;5:63. doi: 10.3389/fbioe.2017.00063 (PMC5653697; doi:10.3389/fbioe.2017.00063)
Supplement: Supplementary file 2 [file Table_1.DOCX]

**Supplementary Table S1: Mean induced and uninduced yEGFP fluorescence intensities for all synTALE/synP pairs. Values are mean values from three independent experiments, with three technical replicates per experiment. Fl, fluorescence; a.u., arbitrary units.**

|  |  | *Uninduced* | | *Induced* | |  |
| --- | --- | --- | --- | --- | --- | --- |
| *SynTALE* | *SynP* | *Mean GFP fl. /cell [a.u.]* | *SD* | *Mean GFP fl. /cell [a.u.]* | *SD* | *Fold change* |
| *SynTALE1* | *1xBS1_fwd* | 3,83 | 0,62 | 411,85 | 159,12 | 107,62 |
|  | *1xBS1_rev* | 3,83 | 0,18 | 320,09 | 68,11 | 83,65 |
|  | *2xBS1_fwd* | 3,84 | 0,22 | 501,94 | 91,46 | 130,71 |
|  | *2xBS1_rev* | 4,09 | 0,28 | 673,51 | 135,77 | 164,68 |
|  | *4xBS1_fwd* | 3,41 | 0,23 | 488,53 | 122,54 | 143,42 |
|  | *4xBS1_m1_fwd* | 3,46 | 0,27 | 425,27 | 34,77 | 122,88 |
|  | *4xBS1_m2_fwd* | 3,54 | 0,52 | 1063,50 | 78,36 | 300,75 |
|  | *4xBS1_m3_fwd* | 3,14 | 0,32 | 68,18 | 37,36 | 21,71 |
|  | *4xBS1_rev* | 3,59 | 0,41 | 1097,25 | 192,46 | 305,76 |
|  | *4xBS1_m1_rev* | 4,74 | 0,62 | 2133,68 | 120,64 | 450,37 |
|  | *4xBS1_m2_rev* | 3,37 | 0,32 | 216,69 | 146,91 | 64,22 |
|  | *4xBS1_m3_rev* | 3,11 | 0,27 | 23,74 | 4,59 | 7,63 |
|  | *no_BS* | 3,82 | 0,55 | 9,93 | 1,49 | 2,60 |
|  | *TDH3p* | 578,54 | 164,92 | 743,07 | 165,70 | 1,28 |
| *SynTALE2* | *1xBS1_fwd* | 3,65 | 0,78 | 270,94 | 17,17 | 74,15 |
|  | *1xBS1_rev* | 3,80 | 0,18 | 353,03 | 11,27 | 93,01 |
|  | *2xBS1_fwd* | 3,70 | 0,28 | 367,57 | 72,30 | 99,33 |
|  | *2xBS1_rev* | 3,89 | 0,24 | 561,31 | 100,61 | 144,41 |
|  | *4xBS1_fwd* | 3,46 | 0,38 | 345,09 | 70,38 | 99,70 |
|  | *4xBS1_m1_fwd* | 2,57 | 0,28 | 122,42 | 12,59 | 47,62 |
|  | *4xBS1_m2_fwd* | 3,53 | 0,39 | 210,98 | 22,09 | 59,72 |
|  | *4xBS1_m3_fwd* | 3,49 | 0,37 | 50,52 | 25,97 | 14,49 |
|  | *4xBS1_rev* | 3,83 | 0,38 | 920,88 | 248,20 | 240,65 |
|  | *4xBS1_m1_rev* | 4,04 | 0,43 | 424,51 | 62,55 | 105,14 |
|  | *4xBS1_m2_rev* | 2,45 | 0,27 | 188,26 | 36,80 | 76,81 |
|  | *4xBS1_m3_rev* | 3,07 | 0,31 | 17,23 | 1,78 | 5,62 |
|  | *no_BS* | 3,73 | 0,60 | 8,92 | 1,72 | 2,39 |
|  | *TDH3p* | 566,29 | 150,02 | 805,55 | 158,06 | 1,42 |
| *SynTALE3* | *1xBS2_fwd* | 4,16 | 0,41 | 5,84 | 0,35 | 1,40 |
|  | *1xBS2_rev* | 4,20 | 0,31 | 76,04 | 16,73 | 18,08 |
|  | *2xBS2_fwd* | 3,74 | 0,87 | 576,62 | 95,50 | 154,25 |
|  | *2xBS2_rev* | 4,66 | 0,83 | 634,03 | 52,08 | 136,14 |
|  | *4xBS2_fwd* | 4,93 | 1,31 | 551,86 | 236,84 | 111,91 |
|  | *4xBS2_m1_fwd* | 5,30 | 1,68 | 350,58 | 50,50 | 66,10 |
|  | *4xBS2_m2_fwd* | 3,23 | 0,37 | 297,24 | 29,40 | 92,04 |
|  | *4xBS2_m3_fwd* | 3,12 | 0,30 | 4,22 | 0,26 | 1,35 |
|  | *4xBS2_rev* | 4,52 | 1,01 | 567,99 | 224,91 | 125,72 |
|  | *4xBS2_m1_rev* | 4,64 | 1,75 | 349,59 | 49,40 | 75,33 |
|  | *4xBS2_m2_rev* | 2,83 | 0,48 | 284,18 | 36,68 | 100,34 |
|  | *4xBS2_m3_rev* | 3,09 | 0,42 | 4,45 | 0,32 | 1,44 |
|  | *no_BS* | 3,51 | 0,45 | 5,30 | 1,46 | 1,51 |
|  | *TDH3p* | 599,62 | 156,94 | 246,15 | 32,87 | 0,41 |
| *SynTALE4* | *1xBS2_fwd* | 4,22 | 0,33 | 67,16 | 12,62 | 15,90 |
|  | *1xBS2_rev* | 4,23 | 0,39 | 28,83 | 9,64 | 6,81 |
|  | *2xBS2_fwd* | 4,64 | 0,37 | 371,10 | 105,16 | 79,90 |
|  | *2xBS2_rev* | 4,61 | 0,55 | 590,57 | 111,55 | 128,13 |
|  | *4xBS2_fwd* | 4,40 | 0,67 | 459,34 | 156,23 | 104,48 |
|  | *4xBS2_m1_fwd* | 6,88 | 2,65 | 766,86 | 66,57 | 111,41 |
|  | *4xBS2_m2_fwd* | 3,13 | 0,36 | 615,17 | 58,44 | 196,23 |
|  | *4xBS2_m3_fwd* | 2,91 | 0,17 | 5,84 | 0,66 | 2,01 |
|  | *4xBS2_rev* | 4,53 | 1,04 | 848,52 | 254,96 | 187,35 |
|  | *4xBS2_m1_rev* | 4,38 | 1,11 | 499,97 | 59,03 | 114,11 |
|  | *4xBS2_m2_rev* | 3,43 | 0,58 | 549,19 | 95,26 | 159,92 |
|  | *4xBS2_m3_rev* | 3,43 | 0,55 | 6,06 | 0,68 | 1,77 |
|  | *no_BS* | 3,75 | 0,48 | 6,27 | 0,74 | 1,67 |
|  | *TDH3p* | 604,81 | 230,94 | 344,59 | 40,90 | 0,57 |
| *SynTALE5* | *1xBS3_fwd* | 4,21 | 0,73 | 6,55 | 0,47 | 1,55 |
|  | *1xBS3_rev* | 4,96 | 1,27 | 7,58 | 1,29 | 1,53 |
|  | *2xBS3_fwd* | 6,37 | 0,78 | 10,69 | 1,31 | 1,68 |
|  | *2xBS3_rev* | 5,70 | 0,94 | 9,78 | 0,68 | 1,72 |
|  | *4xBS3_fwd* | 7,23 | 1,56 | 14,35 | 1,38 | 1,98 |
|  | *4xBS3_rev* | 7,87 | 1,53 | 15,14 | 1,86 | 1,93 |
|  | *no_BS* | 4,39 | 1,56 | 5,64 | 0,20 | 1,28 |
|  | *TDH3p* | 714,67 | 60,39 | 358,59 | 59,05 | 0,50 |
| *SynTALE6* | *1xBS3_fwd* | 3,98 | 0,49 | 6,67 | 0,85 | 1,68 |
|  | *1xBS3_rev* | 18,86 | 2,41 | 15,98 | 1,54 | 0,85 |
|  | *2xBS3_fwd* | 6,43 | 0,73 | 11,17 | 1,21 | 1,74 |
|  | *2xBS3_rev* | 10,76 | 2,15 | 19,56 | 1,92 | 1,82 |
|  | *4xBS3_fwd* | 7,44 | 0,72 | 13,30 | 1,30 | 1,79 |
|  | *4xBS3_rev* | 28,94 | 7,06 | 29,66 | 4,29 | 1,02 |
|  | *no_BS* | 4,20 | 0,59 | 6,01 | 0,70 | 1,43 |
|  | *TDH3p* | 760,73 | 62,99 | 340,18 | 30,89 | 0,45 |
| *SynTALE7* | *1xBS4_fwd* | 4,20 | 0,79 | 296,33 | 11,97 | 70,57 |
|  | *1xBS4_rev* | 6,05 | 1,92 | 278,15 | 18,89 | 45,99 |
|  | *2xBS4_fwd* | 3,91 | 0,75 | 261,79 | 22,46 | 66,87 |
|  | *2xBS4_rev* | 4,07 | 0,80 | 287,63 | 19,09 | 70,66 |
|  | *4xBS4_fwd* | 3,51 | 0,75 | 334,91 | 26,32 | 95,40 |
|  | *4xBS4_rev* | 4,15 | 0,60 | 1665,20 | 107,79 | 400,95 |
|  | *no_BS* | 3,75 | 0,83 | 3,34 | 0,67 | 0,89 |
|  | *TDH3p* | 545,57 | 173,95 | 301,40 | 54,40 | 0,55 |
| *SynTALE8* | *1xBS4_fwd* | 3,84 | 0,80 | 85,98 | 13,22 | 22,40 |
|  | *1xBS4_rev* | 4,02 | 0,88 | 114,25 | 23,72 | 28,41 |
|  | *2xBS4_fwd* | 4,36 | 1,10 | 391,96 | 119,62 | 89,89 |
|  | *2xBS4_rev* | 3,88 | 0,83 | 548,23 | 180,37 | 141,36 |
|  | *4xBS4_fwd* | 3,69 | 0,69 | 386,92 | 132,44 | 104,72 |
|  | *4xBS4_rev* | 3,45 | 0,75 | 1012,19 | 62,70 | 293,36 |
|  | *no_BS* | 3,87 | 0,87 | 5,38 | 0,61 | 1,39 |
|  | *TDH3p* | 479,74 | 150,29 | 319,23 | 46,59 | 0,67 |
| *SynTALE9* | *1xBS5_fwd* | 3,69 | 0,91 | 15,81 | 1,71 | 4,29 |
|  | *1xBS5_rev* | 4,52 | 0,91 | 29,12 | 9,60 | 6,44 |
|  | *2xBS5_fwd* | 3,58 | 0,74 | 34,62 | 12,09 | 9,67 |
|  | *2xBS5_rev* | 3,74 | 0,67 | 43,72 | 12,68 | 11,68 |
|  | *4xBS5_fwd* | 3,17 | 0,61 | 50,37 | 15,99 | 15,90 |
|  | *4xBS5_rev* | 3,38 | 0,85 | 94,04 | 26,61 | 27,85 |
|  | *no_BS* | 4,76 | 1,08 | 8,97 | 0,80 | 1,88 |
|  | *TDH3p* | 1096,96 | 364,93 | 1532,75 | 202,81 | 1,40 |
| *SynTALE10* | *1xBS5_fwd* | 3,61 | 0,64 | 18,43 | 4,71 | 5,10 |
|  | *1xBS5_rev* | 4,47 | 0,87 | 23,75 | 5,40 | 5,32 |
|  | *2xBS5_fwd* | 3,65 | 0,85 | 27,24 | 10,27 | 7,46 |
|  | *2xBS5_rev* | 3,89 | 0,89 | 42,30 | 14,17 | 10,88 |
|  | *4xBS5_fwd* | 3,75 | 1,08 | 36,57 | 19,05 | 9,74 |
|  | *4xBS5_rev* | 3,31 | 0,91 | 41,74 | 15,26 | 12,61 |
|  | *no_BS* | 3,81 | 0,84 | 5,87 | 0,53 | 1,54 |
|  | *TDH3p* | 495,85 | 163,70 | 664,01 | 75,68 | 1,34 |
| *SynTALE11* | *2xBS11_fwd* | 5,11 | 1,36 | 290,47 | 28,35 | 56,88 |
|  | *4xBS11_fwd* | 3,26 | 0,32 | 347,06 | 29,67 | 106,49 |
|  | *8xBS11_fwd* | 2,41 | 0,72 | 493,04 | 85,68 | 204,24 |
|  | *16xBS11_fwd* | 3,59 | 1,66 | 786,33 | 115,26 | 218,81 |
|  | *no_BS* | 2,21 | 0,08 | 2,24 | 0,17 | 1,01 |
|  | *TDH3p* | 3697,84 | 359,36 | 4095,66 | 648,18 | 1,11 |
| *SynTALE12* | *2xBS12_fwd* | 3,93 | 0,27 | 7,36 | 0,41 | 1,87 |
|  | *4xBS12_fwd* | 2,10 | 0,24 | 2,44 | 0,37 | 1,16 |
|  | *8xBS12_fwd* | 5,33 | 3,89 | 2,59 | 0,58 | 0,49 |
|  | *16xBS12_fwd* | 6,48 | 3,84 | 2,68 | 0,61 | 0,41 |
|  | *no_BS* | 3,87 | 0,35 | 2,83 | 0,19 | 0,73 |
|  | *TDH3p* | 619,30 | 180,10 | 293,23 | 41,26 | 0,47 |
| *SynTALE13* | *2xBS13_fwd* | 58,18 | 31,71 | 502,85 | 51,03 | 8,64 |
|  | *4xBS13_fwd* | 10,24 | 1,30 | 38,15 | 1,31 | 3,73 |
|  | *8xBS13_fwd* | 36,43 | 3,10 | 541,81 | 50,36 | 14,87 |
|  | *16xBS13_fwd* | 25,99 | 2,82 | 557,78 | 32,62 | 21,46 |
|  | *no_BS* | 3,35 | 0,22 | 2,14 | 0,31 | 0,64 |
|  | *TDH3p* | 610,53 | 146,20 | 266,81 | 29,35 | 0,44 |
| *SynTALE14* | *2xBS14_fwd* | 7,51 | 2,11 | 241,01 | 18,25 | 32,09 |
|  | *4xBS14_fwd* | 5,52 | 1,37 | 280,81 | 18,81 | 50,85 |
|  | *8xBS14_fwd* | 5,25 | 0,61 | 357,67 | 49,64 | 68,11 |
|  | *16xBS14_fwd* | 4,18 | 0,62 | 460,32 | 58,03 | 110,07 |
|  | *no_BS* | 3,09 | 0,23 | 2,49 | 0,10 | 0,80 |
|  | *TDH3p* | 624,61 | 175,77 | 297,17 | 43,38 | 0,48 |
| *SynTALE15* | *2xBS15_fwd* | 3,43 | 0,36 | 224,26 | 17,19 | 65,46 |
|  | *4xBS15_fwd* | 4,36 | 0,71 | 514,10 | 20,73 | 117,82 |
|  | *8xBS15_fwd* | 2,80 | 1,46 | 712,55 | 100,92 | 254,35 |
|  | *16xBS15_fwd* | 3,24 | 1,69 | 414,92 | 59,59 | 128,03 |
|  | *no_BS* | 3,00 | 0,20 | 2,47 | 0,14 | 0,83 |
|  | *TDH3p* | 584,31 | 142,32 | 287,19 | 30,24 | 0,49 |
