# Supplementary material for: Synthetic Promoters and Transcription Factors for Heterologous Protein Expression in Saccharomyces cerevisiae
Source: Front Bioeng Biotechnol. 2017 Oct 19;5:63. doi: 10.3389/fbioe.2017.00063 (PMC5653697; doi:10.3389/fbioe.2017.00063)
Supplement: Supplementary file 3 [file Table_2.DOCX]

**Supplementary Table S2: Mean induced and uninduced GFP fluorescence intensities for all dCas9-VP64 - synP pairs. Values are mean values from three independent experiments, with three technical replicates per experiment. Values marked with an asterisk are derived from two independent experiments. Fl, fluorescence; a.u., arbitrary units.**

|  | *Unspecific guide* | | *Specific guide* | |  |
| --- | --- | --- | --- | --- | --- |
| *SynP* | *Mean GFP fl. / cell [a.u.]* | *SD* | *Mean GFP fl. / cell [a.u.]* | *SD* | *Fold change* |
| *2xBS11_fwd* | 8,58 | 1,06 | 30,52 | 2,33 | 3,56 |
| *4xBS11_fwd* | 5,60 | 0,70 | 50,14 | 13,54 | 8,95 |
| *8xBS11_fwd* | 5,43 | 0,56 | 10,27 | 0,89 | 1,89 |
| *16xBS11_fwd* | 3,24^*^ | 0,43 | 2,84^*^ | 0,30 | 0,88 |
| *2xBS12_fwd* | 6,34 | 1,26 | 92,88 | 6,76 | 14,66 |
| *4xBS12_fwd* | 2,82 | 0,57 | 12,02 | 5,35 | 4,26 |
| *8xBS12_fwd* | 3,95 | 0,31 | 3,70 | 0,20 | 0,94 |
| *16xBS12_fwd* | 3,00^*^ | 0,14 | 2,51^*^ | 0,18 | 0,83 |
| *2xBS13_fwd* | 17,23 | 1,16 | 89,44 | 4,25 | 5,19 |
| *4xBS13_fwd* | 16,48 | 1,23 | 107,84 | 7,14 | 6,54 |
| *8xBS13_fwd* | 8,93 | 1,88 | 160,75 | 10,69 | 17,99 |
| *16xBS13_fwd* | 76,36^*^ | 14,47 | 29,43^*^ | 18,42 | 0,39 |
| *2xBS14_fwd* | 17,39 | 1,53 | 38,24 | 4,53 | 2,20 |
| *4xBS14_fwd* | 7,99 | 0,60 | 18,68 | 2,23 | 2,34 |
| *8xBS14_fwd* | 5,26 | 0,72 | 227,91 | 36,46 | 43,31 |
| *16xBS14_fwd* | 9,16^*^ | 3,21 | 4,54^*^ | 1,07 | 0,50 |
| *2xBS15_fwd* | 7,47 | 0,68 | 20,81 | 1,69 | 2,78 |
| *4xBS15_fwd* | 6,46 | 1,66 | 62,88 | 3,46 | 9,73 |
| *8xBS15_fwd* | 7,95 | 2,68 | 14,67 | 9,10 | 1,85 |
| *16xBS15_fwd* | 3,36^*^ | 0,96 | 3,79^*^ | 0,41 | 1,13 |
