# Supplementary material for: Synthetic Promoters and Transcription Factors for Heterologous Protein Expression in Saccharomyces cerevisiae
Source: Front Bioeng Biotechnol. 2017 Oct 19;5:63. doi: 10.3389/fbioe.2017.00063 (PMC5653697; doi:10.3389/fbioe.2017.00063)
Supplement: Supplementary file 4 [file Table_3.DOCX]

**Supplementary Table S3:** I**nduced and uninduced mean GFP fluorescence intensities for all synTF/synP pairs with fold induction ≥ 1.5. Pairs are sorted by induced yEGFP fluorescence output. a.u., arbitrary units.**

| *#* | *SynTF* | *SynP* | *Uninduced GFP fluorescence [a.u.]* | *SD* | *Induced GFP fluorescence [a.u.]* | *SD* | *Fold change* |
| --- | --- | --- | --- | --- | --- | --- | --- |
| 1 | SynTALE4 | 4xBS2_m3_fwd | 2,9 | 0,2 | 5,8 | 0,7 | 2,0 |
| 2 | SynTALE4 | 4xBS2_m3_rev | 3,4 | 0,6 | 6,1 | 0,7 | 1,8 |
| 3 | SynTALE5 | 1xBS3_fwd | 4,2 | 0,7 | 6,6 | 0,5 | 1,6 |
| 4 | SynTALE6 | 1xBS3_fwd | 4,0 | 0,5 | 6,7 | 0,8 | 1,7 |
| 5 | SynTALE12 | 2xBS12_fwd | 3,9 | 0,3 | 7,4 | 0,4 | 1,9 |
| 6 | SynTALE5 | 1xBS3_rev | 5,0 | 1,3 | 7,6 | 1,3 | 1,5 |
| 7 | SynTALE5 | 2xBS3_rev | 5,7 | 0,9 | 9,8 | 0,7 | 1,7 |
| 8 | dCas9 | 8xBS11_fwd | 5,4 | 0,6 | 10,3 | 0,9 | 1,9 |
| 9 | SynTALE5 | 2xBS3_fwd | 6,4 | 0,8 | 10,7 | 1,3 | 1,7 |
| 10 | SynTALE6 | 2xBS3_fwd | 6,4 | 0,7 | 11,2 | 1,2 | 1,7 |
| 11 | dCas9 | 4xBS12_fwd | 2,8 | 0,6 | 12,0 | 5,4 | 4,3 |
| 12 | SynTALE6 | 4xBS3_fwd | 7,4 | 0,7 | 13,3 | 1,3 | 1,8 |
| 13 | SynTALE5 | 4xBS3_fwd | 7,2 | 1,6 | 14,3 | 1,4 | 2,0 |
| 14 | dCas9 | 8xBS15_fwd | 7,9 | 2,7 | 14,7 | 9,1 | 1,8 |
| 15 | SynTALE5 | 4xBS3_rev | 7,9 | 1,5 | 15,1 | 1,9 | 1,9 |
| 16 | SynTALE9 | 1xBS5_fwd | 3,7 | 0,9 | 15,8 | 1,7 | 4,3 |
| 17 | SynTALE2 | 4xBS1_m3_rev | 3,1 | 0,3 | 17,2 | 1,8 | 5,6 |
| 18 | SynTALE10 | 1xBS5_fwd | 3,6 | 0,6 | 18,4 | 4,7 | 5,1 |
| 19 | dCas9 | 4xBS14_fwd | 8,0 | 0,6 | 18,7 | 2,2 | 2,3 |
| 20 | SynTALE6 | 2xBS3_rev | 10,8 | 2,1 | 19,6 | 1,9 | 1,8 |
| 21 | dCas9 | 2xBS15_fwd | 7,5 | 0,7 | 20,8 | 1,7 | 2,8 |
| 22 | SynTALE1 | 4xBS1_m3_rev | 3,1 | 0,3 | 23,7 | 4,6 | 7,6 |
| 23 | SynTALE10 | 1xBS5_rev | 4,5 | 0,9 | 23,7 | 5,4 | 5,3 |
| 24 | SynTALE10 | 2xBS5_fwd | 3,7 | 0,8 | 27,2 | 10,3 | 7,5 |
| 25 | SynTALE4 | 1xBS2_rev | 4,2 | 0,4 | 28,8 | 9,6 | 6,8 |
| 26 | SynTALE9 | 1xBS5_rev | 4,5 | 0,9 | 29,1 | 9,6 | 6,4 |
| 27 | dCas9 | 2xBS11_fwd | 8,6 | 1,1 | 30,5 | 2,3 | 3,6 |
| 28 | SynTALE9 | 2xBS5_fwd | 3,6 | 0,7 | 34,6 | 12,1 | 9,7 |
| 29 | SynTALE10 | 4xBS5_fwd | 3,8 | 1,1 | 36,6 | 19,1 | 9,7 |
| 30 | SynTALE13 | 4xBS13_fwd | 10,2 | 1,3 | 38,2 | 1,3 | 3,7 |
| 31 | dCas9 | 2xBS14_fwd | 17,4 | 1,5 | 38,2 | 4,5 | 2,2 |
| 32 | SynTALE10 | 4xBS5_rev | 3,3 | 0,9 | 41,7 | 15,3 | 12,6 |
| 33 | SynTALE10 | 2xBS5_rev | 3,9 | 0,9 | 42,3 | 14,2 | 10,9 |
| 34 | SynTALE9 | 2xBS5_rev | 3,7 | 0,7 | 43,7 | 12,7 | 11,7 |
| 35 | dCas9 | 4xBS11_fwd | 5,6 | 0,7 | 50,1 | 13,5 | 9,0 |
| 36 | SynTALE9 | 4xBS5_fwd | 3,2 | 0,6 | 50,4 | 16,0 | 15,9 |
| 37 | SynTALE2 | 4xBS1_m3_fwd | 3,5 | 0,4 | 50,5 | 26,0 | 14,5 |
| 38 | dCas9 | 4xBS15_fwd | 6,5 | 1,7 | 62,9 | 3,5 | 9,7 |
| 39 | SynTALE4 | 1xBS2_fwd | 4,2 | 0,3 | 67,2 | 12,6 | 15,9 |
| 40 | SynTALE1 | 4xBS1_m3_fwd | 3,1 | 0,3 | 68,2 | 37,4 | 21,7 |
| 41 | SynTALE3 | 1xBS2_rev | 4,2 | 0,3 | 76,0 | 16,7 | 18,1 |
| 42 | SynTALE8 | 1xBS4_fwd | 3,8 | 0,8 | 86,0 | 13,2 | 22,4 |
| 43 | dCas9 | 2xBS13_fwd | 17,2 | 1,2 | 89,4 | 4,3 | 5,2 |
| 44 | dCas9 | 2xBS12_fwd | 6,3 | 1,3 | 92,9 | 6,8 | 14,7 |
| 45 | SynTALE9 | 4xBS5_rev | 3,4 | 0,9 | 94,0 | 26,6 | 27,8 |
| 46 | dCas9 | 4xBS13_fwd | 16,5 | 1,2 | 107,8 | 7,1 | 6,5 |
| 47 | SynTALE8 | 1xBS4_rev | 4,0 | 0,9 | 114,2 | 23,7 | 28,4 |
| 48 | SynTALE2 | 4xBS1_m1_fwd | 2,6 | 0,3 | 122,4 | 12,6 | 47,6 |
| 49 | dCas9 | 8xBS13_fwd | 8,9 | 1,9 | 160,7 | 10,7 | 18,0 |
| 50 | SynTALE2 | 4xBS1_m2_rev | 2,5 | 0,3 | 188,3 | 36,8 | 76,8 |
| 51 | SynTALE2 | 4xBS1_m2_fwd | 3,5 | 0,4 | 211,0 | 22,1 | 59,7 |
| 52 | SynTALE1 | 4xBS1_m2_rev | 3,4 | 0,3 | 216,7 | 146,9 | 64,2 |
| 53 | SynTALE15 | 2xBS15_fwd | 3,4 | 0,4 | 224,3 | 17,2 | 65,5 |
| 54 | dCas9 | 8xBS14_fwd | 5,3 | 0,7 | 227,9 | 36,5 | 43,3 |
| 55 | SynTALE14 | 2xBS14_fwd | 7,5 | 2,1 | 241,0 | 18,3 | 32,1 |
| 56 | SynTALE7 | 2xBS4_fwd | 3,9 | 0,7 | 261,8 | 22,5 | 66,9 |
| 57 | SynTALE2 | 1xBS1_fwd | 3,7 | 0,8 | 270,9 | 17,2 | 74,2 |
| 58 | SynTALE7 | 1xBS4_rev | 6,0 | 1,9 | 278,2 | 18,9 | 46,0 |
| 59 | SynTALE14 | 4xBS14_fwd | 5,5 | 1,4 | 280,8 | 18,8 | 50,8 |
| 60 | SynTALE3 | 4xBS2_m2_rev | 2,8 | 0,5 | 284,2 | 36,7 | 100,3 |
| 61 | SynTALE7 | 2xBS4_rev | 4,1 | 0,8 | 287,6 | 19,1 | 70,7 |
| 62 | SynTALE11 | 2xBS11_fwd | 5,1 | 1,4 | 290,5 | 28,3 | 56,9 |
| 63 | SynTALE7 | 1xBS4_fwd | 4,2 | 0,8 | 296,3 | 12,0 | 70,6 |
| 64 | SynTALE3 | 4xBS2_m2_fwd | 3,2 | 0,4 | 297,2 | 29,4 | 92,0 |
| 65 | SynTALE1 | 1xBS1_rev | 3,8 | 0,2 | 320,1 | 68,1 | 83,7 |
| 66 | SynTALE7 | 4xBS4_fwd | 3,5 | 0,8 | 334,9 | 26,3 | 95,4 |
| 67 | SynTALE2 | 4xBS1_fwd | 3,5 | 0,4 | 345,1 | 70,4 | 99,7 |
| 68 | SynTALE11 | 4xBS11_fwd | 3,3 | 0,3 | 347,1 | 29,7 | 106,5 |
| 69 | SynTALE3 | 4xBS2_m1_rev | 4,6 | 1,7 | 349,6 | 49,4 | 75,3 |
| 70 | SynTALE3 | 4xBS2_m1_fwd | 5,3 | 1,7 | 350,6 | 50,5 | 66,1 |
| 71 | SynTALE2 | 1xBS1_rev | 3,8 | 0,2 | 353,0 | 11,3 | 93,0 |
| 72 | SynTALE14 | 8xBS14_fwd | 5,3 | 0,6 | 357,7 | 49,6 | 68,1 |
| 73 | SynTALE2 | 2xBS1_fwd | 3,7 | 0,3 | 367,6 | 72,3 | 99,3 |
| 74 | SynTALE4 | 2xBS2_fwd | 4,6 | 0,4 | 371,1 | 105,2 | 79,9 |
| 75 | SynTALE8 | 4xBS4_fwd | 3,7 | 0,7 | 386,9 | 132,4 | 104,7 |
| 76 | SynTALE8 | 2xBS4_fwd | 4,4 | 1,1 | 392,0 | 119,6 | 89,9 |
| 77 | SynTALE1 | 1xBS1_fwd | 3,8 | 0,6 | 411,9 | 159,1 | 107,6 |
| 78 | SynTALE15 | 16xBS15_fwd | 3,2 | 1,7 | 414,9 | 59,6 | 128,0 |
| 79 | SynTALE2 | 4xBS1_m1_rev | 4,0 | 0,4 | 424,5 | 62,5 | 105,1 |
| 80 | SynTALE1 | 4xBS1_m1_fwd | 3,5 | 0,3 | 425,3 | 34,8 | 122,9 |
| 81 | SynTALE4 | 4xBS2_fwd | 4,4 | 0,7 | 459,3 | 156,2 | 104,5 |
| 82 | SynTALE14 | 16xBS14_fwd | 4,2 | 0,6 | 460,3 | 58,0 | 110,1 |
| 83 | SynTALE1 | 4xBS1_fwd | 3,4 | 0,2 | 488,5 | 122,5 | 143,4 |
| 84 | SynTALE11 | 8xBS11_fwd | 2,4 | 0,7 | 493,0 | 85,7 | 204,2 |
| 85 | SynTALE4 | 4xBS2_m1_rev | 4,4 | 1,1 | 500,0 | 59,0 | 114,1 |
| 86 | SynTALE1 | 2xBS1_fwd | 3,8 | 0,2 | 501,9 | 91,5 | 130,7 |
| 87 | SynTALE13 | 2xBS13_fwd | 58,2 | 31,7 | 502,9 | 51,0 | 8,6 |
| 88 | SynTALE15 | 4xBS15_fwd | 4,4 | 0,7 | 514,1 | 20,7 | 117,8 |
| 89 | SynTALE13 | 8xBS13_fwd | 36,4 | 3,1 | 541,8 | 50,4 | 14,9 |
| 90 | SynTALE8 | 2xBS4_rev | 3,9 | 0,8 | 548,2 | 180,4 | 141,4 |
| 91 | SynTALE4 | 4xBS2_m2_rev | 3,4 | 0,6 | 549,2 | 95,3 | 159,9 |
| 92 | SynTALE3 | 4xBS2_fwd | 4,9 | 1,3 | 551,9 | 236,8 | 111,9 |
| 93 | SynTALE13 | 16xBS13_fwd | 26,0 | 2,8 | 557,8 | 32,6 | 21,5 |
| 94 | SynTALE2 | 2xBS1_rev | 3,9 | 0,2 | 561,3 | 100,6 | 144,4 |
| 95 | SynTALE3 | 4xBS2_rev | 4,5 | 1,0 | 568,0 | 224,9 | 125,7 |
| 96 | SynTALE3 | 2xBS2_fwd | 3,7 | 0,9 | 576,6 | 95,5 | 154,3 |
| 97 | SynTALE4 | 2xBS2_rev | 4,6 | 0,5 | 590,6 | 111,6 | 128,1 |
| 98 | SynTALE4 | 4xBS2_m2_fwd | 3,1 | 0,4 | 615,2 | 58,4 | 196,2 |
| 99 | SynTALE3 | 2xBS2_rev | 4,7 | 0,8 | 634,0 | 52,1 | 136,1 |
| 100 | SynTALE1 | 2xBS1_rev | 4,1 | 0,3 | 673,5 | 135,8 | 164,7 |
| 101 | SynTALE15 | 8xBS15_fwd | 2,8 | 1,5 | 712,6 | 100,9 | 254,3 |
| 102 | SynTALE4 | 4xBS2_m1_fwd | 6,9 | 2,6 | 766,9 | 66,6 | 111,4 |
| 103 | SynTALE11 | 16xBS11_fwd | 3,6 | 1,7 | 786,3 | 115,3 | 218,8 |
| 104 | SynTALE4 | 4xBS2_rev | 4,5 | 1,0 | 848,5 | 255,0 | 187,4 |
| 105 | SynTALE2 | 4xBS1_rev | 3,8 | 0,4 | 920,9 | 248,2 | 240,6 |
| 106 | SynTALE8 | 4xBS4_rev | 3,5 | 0,8 | 1012,2 | 62,7 | 293,4 |
| 107 | SynTALE1 | 4xBS1_m2_fwd | 3,5 | 0,5 | 1063,5 | 78,4 | 300,7 |
| 108 | SynTALE1 | 4xBS1_rev | 3,6 | 0,4 | 1097,2 | 192,5 | 305,8 |
| 109 | SynTALE7 | 4xBS4_rev | 4,2 | 0,6 | 1665,2 | 107,8 | 400,9 |
| 110 | SynTALE1 | 4xBS1_m1_rev | 4,7 | 0,6 | 2133,7 | 120,6 | 450,4 |
