# Supplementary material for: Synthetic Promoters and Transcription Factors for Heterologous Protein Expression in Saccharomyces cerevisiae
Source: Front Bioeng Biotechnol. 2017 Oct 19;5:63. doi: 10.3389/fbioe.2017.00063 (PMC5653697; doi:10.3389/fbioe.2017.00063)
Supplement: Supplementary file 5 [file Table_4.DOCX]

Supplementary Table S4: Oligonucleotides used for cloning of binding site arrays. Mutated nucleotides are highlighted in red in the upper sequence.

| *Oligo name* | *Nucleotide sequence* |
| --- | --- |
| BS1 upper oligo | CGCGTTCTATAAGATCTTGTGTGCGCTAGCTCTATAAGATCTTGTGTGCGCTAGCTCTATAAGATCTTGTGTGCGCTAGCTCTATAAGATCTTGTGTGCA |
| BS1 lower oligo | CGCGTGCACACAAGATCTTATAGAGCTAGCGCACACAAGATCTTATAGAGCTAGCGCACACAAGATCTTATAGAGCTAGCGCACACAAGATCTTATAGAA |
| BS2 upper oligo | CGCGTTAGTCAAAGTCATTCGTAAGCTAGCTAGTCAAAGTCATTCGTAAGCTAGCTAGTCAAAGTCATTCGTAAGCTAGCTAGTCAAAGTCATTCGTAAA |
| BS2 lower oligo | CGCGTTTACGAATGACTTTGACTAGCTAGCTTACGAATGACTTTGACTAGCTAGCTTACGAATGACTTTGACTAGCTAGCTTACGAATGACTTTGACTAA |
| BS3 upper oligo | CGCGTTGACCAAGCACCAATTAAAGCTAGCTGACCAAGCACCAATTAAAGCTAGCTGACCAAGCACCAATTAAAGCTAGCTGACCAAGCACCAATTAAAA |
| BS3 lower oligo | CGCGTTTTAATTGGTGCTTGGTCAGCTAGCTTTAATTGGTGCTTGGTCAGCTAGCTTTAATTGGTGCTTGGTCAGCTAGCTTTAATTGGTGCTTGGTCAA |
| BS4 upper oligo | CGCGTTAATCAATAAATAGATAAAGCTAGCTAATCAATAAATAGATAAAGCTAGCTAATCAATAAATAGATAAAGCTAGCTAATCAATAAATAGATAAAA |
| BS4 lower oligo | CGCGTTTTATCTATTTATTGATTAGCTAGCTTTATCTATTTATTGATTAGCTAGCTTTATCTATTTATTGATTAGCTAGCTTTATCTATTTATTGATTAA |
| BS5 upper oligo | CGCGTTATATATATGTATAGAGAAGCTAGCTATATATATGTATAGAGAAGCTAGCTATATATATGTATAGAGAAGCTAGCTATATATATGTATAGAGAAA |
| BS5 lower oligo | CGCGTTTCTCTATACATATATATAGCTAGCTTCTCTATACATATATATAGCTAGCTTCTCTATACATATATATAGCTAGCTTCTCTATACATATATATAA |
| BS1_m1 upper oligo | CGCGTTCTATAAAATCTTGTGTGCGCTAGCTCTATAAAATCTTGTGTGCGCTAGCTCTATAAAATCTTGTGTGCGCTAGCTCTATAAAATCTTGTGTGCA |
| BS1_m1 lower oligo | CGCGTGCACACAAGATTTTATAGAGCTAGCGCACACAAGATTTTATAGAGCTAGCGCACACAAGATTTTATAGAGCTAGCGCACACAAGATTTTATAGAA |
| BS1_m2 upper oligo | CGCGTTCTATAAAATCTTATGTGCGCTAGCTCTATAAAATCTTATGTGCGCTAGCTCTATAAAATCTTATGTGCGCTAGCTCTATAAAATCTTATGTGCA |
| BS1_m2 lower oligo | CGCGTGCACATAAGATTTTATAGAGCTAGCGCACATAAGATTTTATAGAGCTAGCGCACATAAGATTTTATAGAGCTAGCGCACATAAGATTTTATAGAA |
| BS1_m3 upper oligo | CGCGTTCTATAAAATCTTATGTACGCTAGCTCTATAAAATCTTATGTACGCTAGCTCTATAAAATCTTATGTACGCTAGCTCTATAAAATCTTATGTACA |
| BS1_m3 lower oligo | CGCGTGTACATAAGATTTTATAGAGCTAGCGTACATAAGATTTTATAGAGCTAGCGTACATAAGATTTTATAGAGCTAGCGTACATAAGATTTTATAGAA |
| BS2_m1 upper oligo | CGCGTTAATCAAAGTCATTCGTAAGCTAGCTAATCAAAGTCATTCGTAAGCTAGCTAATCAAAGTCATTCGTAAGCTAGCTAATCAAAGTCATTCGTAAA |
| BS2_m1 lower oligo | CGCGTTTACGAATGACTTTGATTAGCTAGCTTACGAATGACTTTGATTAGCTAGCTTACGAATGACTTTGATTAGCTAGCTTACGAATGACTTTGATTAA |
| BS2_m2 upper oligo | CGCGTTAATCAAAATCATTCGTAAGCTAGCTAATCAAAATCATTCGTAAGCTAGCTAATCAAAATCATTCGTAAGCTAGCTAATCAAAATCATTCGTAAA |
| BS2_m2 lower oligo | CGCGTTTACGAATGATTTTGATTAGCTAGCTTACGAATGATTTTGATTAGCTAGCTTACGAATGATTTTGATTAGCTAGCTTACGAATGATTTTGATTAA |
| BS2_m3 upper oligo | CGCGTTAATCAAAATCATTCATAAGCTAGCTAATCAAAATCATTCATAAGCTAGCTAATCAAAATCATTCATAAGCTAGCTAATCAAAATCATTCATAAA |
| BS2_m3 lower oligo | CGCGTTTATGAATGATTTTGATTAGCTAGCTTATGAATGATTTTGATTAGCTAGCTTATGAATGATTTTGATTAGCTAGCTTATGAATGATTTTGATTAA |
| BS11 upper oligo | AGCTTGCGATGTGCGGCCGCGCTAGCTGAAATGCTGACCATGAATTTGGGCTAGCTGAAATGCTGACCATGAATTTGGGCTAGCGAGACGACGCGTG |
| BS11 lower oligo | GATCCACGCGTCGTCTCGCTAGCCCAAATTCATGGTCAGCATTTCAGCTAGCCCAAATTCATGGTCAGCATTTCAGCTAGCGCGGCCGCACATCGCA |
| BS12 upper oligo | AGCTTGCGATGTGCGGCCGCGCTAGCTAGACGATAGCTCAGGGAGATGGGCTAGCTAGACGATAGCTCAGGGAGATGGGCTAGCGAGACGACGCGTG |
| BS12 lower oligo | GATCCACGCGTCGTCTCGCTAGCCCATCTCCCTGAGCTATCGTCTAGCTAGCCCATCTCCCTGAGCTATCGTCTAGCTAGCGCGGCCGCACATCGCA |
| BS13 upper oligo | AGCTTGCGATGTGCGGCCGCGCTAGCTGTTCTCGAACGGAGAGATATGGGCTAGCTGTTCTCGAACGGAGAGATATGGGCTAGCGAGACGACGCGTG |
| BS12 lower oligo | GATCCACGCGTCGTCTCGCTAGCCCATATCTCTCCGTTCGAGAACAGCTAGCCCATATCTCTCCGTTCGAGAACAGCTAGCGCGGCCGCACATCGCA |
| BS14 upper oligo | AGCTTGCGATGTGCGGCCGCGCTAGCTCCTCTCTGTCGTCGCTAACTGGGCTAGCTCCTCTCTGTCGTCGCTAACTGGGCTAGCGAGACGACGCGTG |
| BS14 lower oligo | GATCCACGCGTCGTCTCGCTAGCCCAGTTAGCGACGACAGAGAGGAGCTAGCCCAGTTAGCGACGACAGAGAGGAGCTAGCGCGGCCGCACATCGCA |
| BS15 upper oligo | AGCTTGCGATGTGCGGCCGCGCTAGCTTGTAAGTACTTAATCTCATTGGGCTAGCTTGTAAGTACTTAATCTCATTGGGCTAGCGAGACGACGCGTG |
| BS15 lower oligo | GATCCACGCGTCGTCTCGCTAGCCCAATGAGATTAAGTACTTACAAGCTAGCCCAATGAGATTAAGTACTTACAAGCTAGCGCGGCCGCACATCGCA |
