# Supplementary material for: Synthetic Promoters and Transcription Factors for Heterologous Protein Expression in Saccharomyces cerevisiae
Source: Front Bioeng Biotechnol. 2017 Oct 19;5:63. doi: 10.3389/fbioe.2017.00063 (PMC5653697; doi:10.3389/fbioe.2017.00063)
Supplement: Supplementary file 7 [file Image_1.PDF]

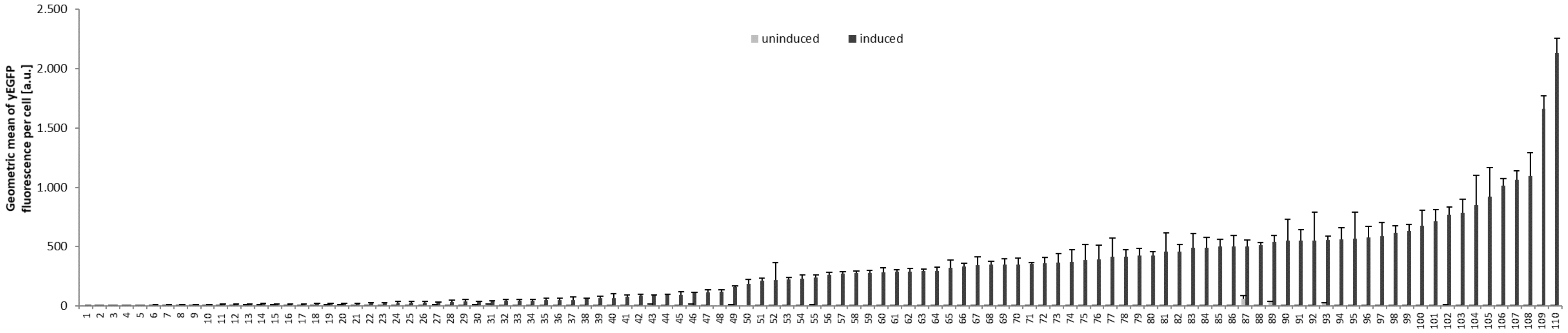

Supplementary Figure S1: Induced and uninduced mean yEGFP fluorescence intensities for all synTF/synP pairs with fold induction ≥ 1.5. synTF/synP pairs are designated according to Supplementary Table S3.
