# Supplementary figures and images for: Synthetic Promoters and Transcription Factors for Heterologous Protein Expression in Saccharomyces cerevisiae
Source: Front Bioeng Biotechnol. 2017 Oct 19;5:63. doi: 10.3389/fbioe.2017.00063 (PMC5653697; doi:10.3389/fbioe.2017.00063)

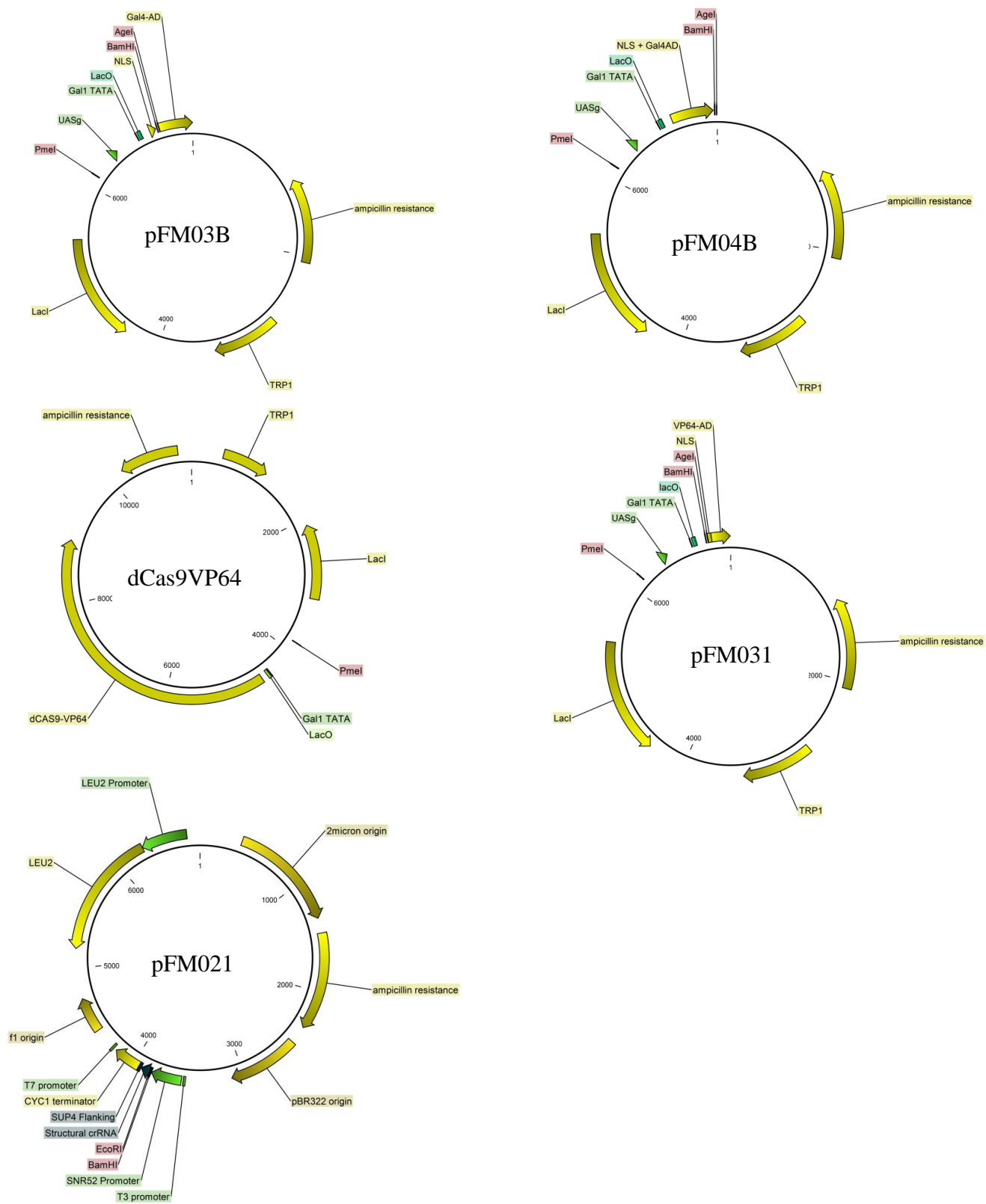

**Supplementary Figure S2:** Maps of plasmids used for synTF and sgRNA expression.

Supplement: Supplementary file 8 [file Image_2.PDF]

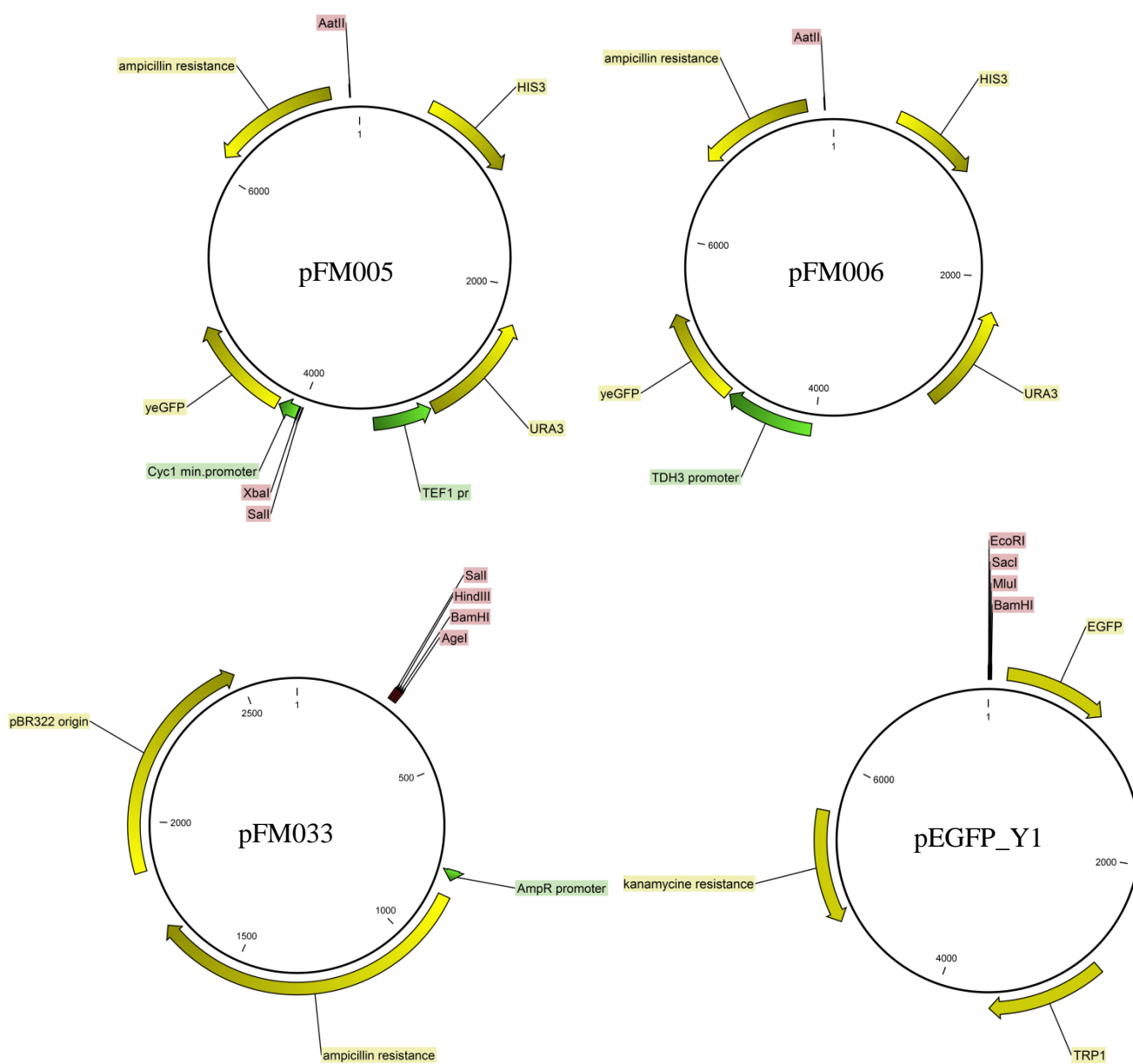

**Supplementary Figure S3:** Maps of reporter plasmids and synP cloning vectors.

Supplement: Supplementary file 9 [file Image_3.PDF]

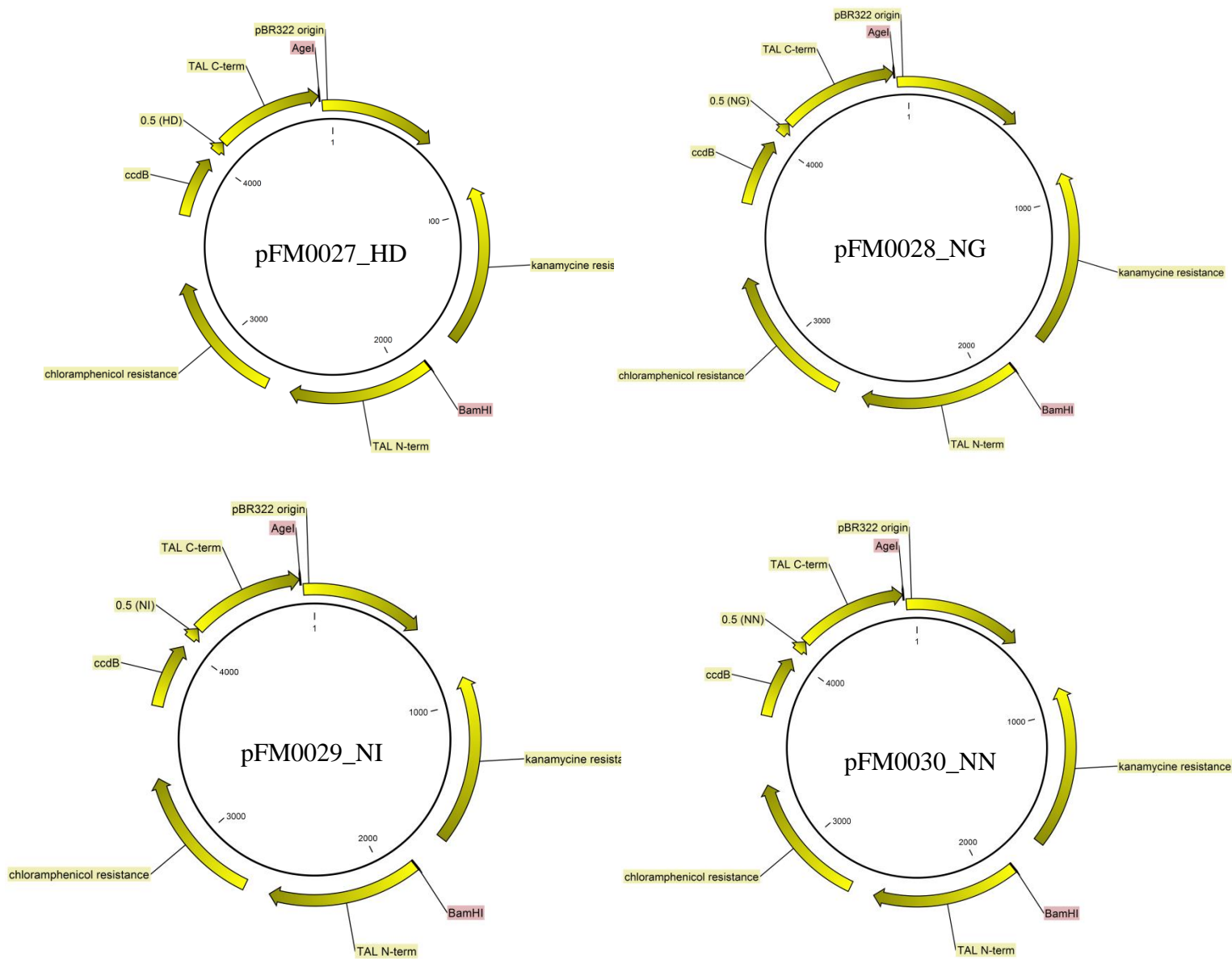

**Supplementary Figure S4:** Maps of plasmids used for synTALE assembly.

Supplement: Supplementary file 10 [file Image_4.PDF]

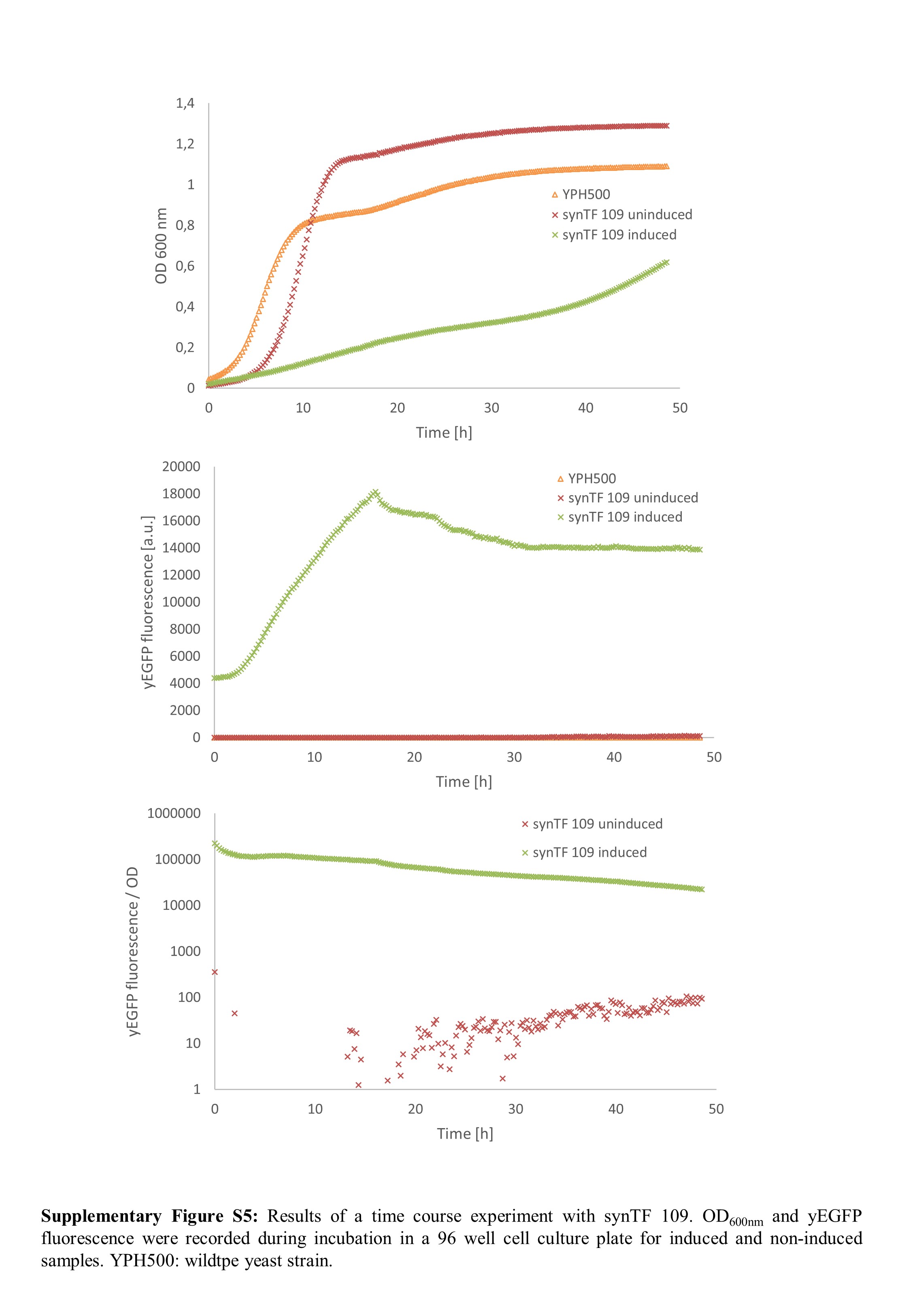

Supplement: Supplementary file 11 [file Image_5.JPEG]

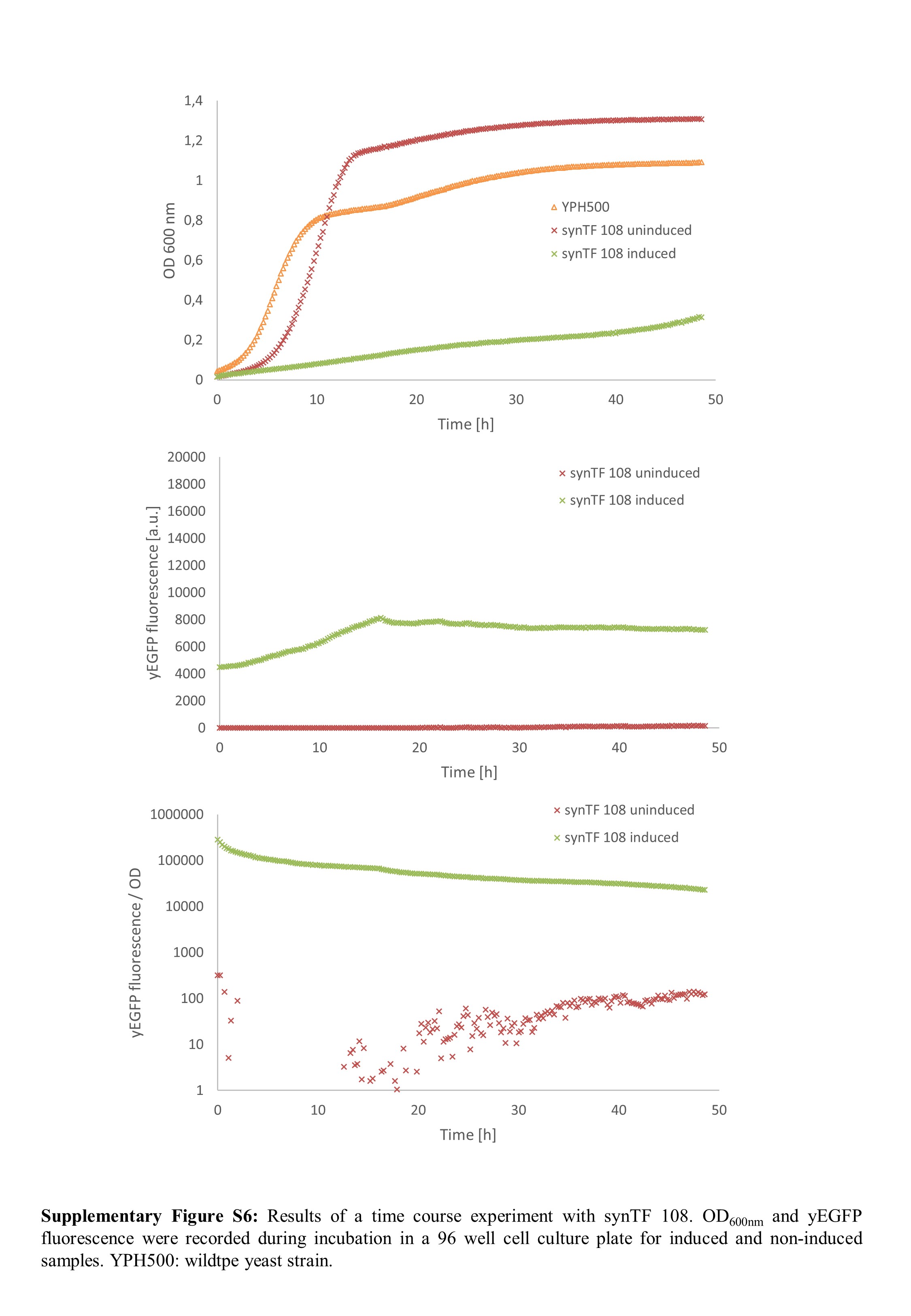

Supplement: Supplementary file 12 [file Image_6.JPEG]

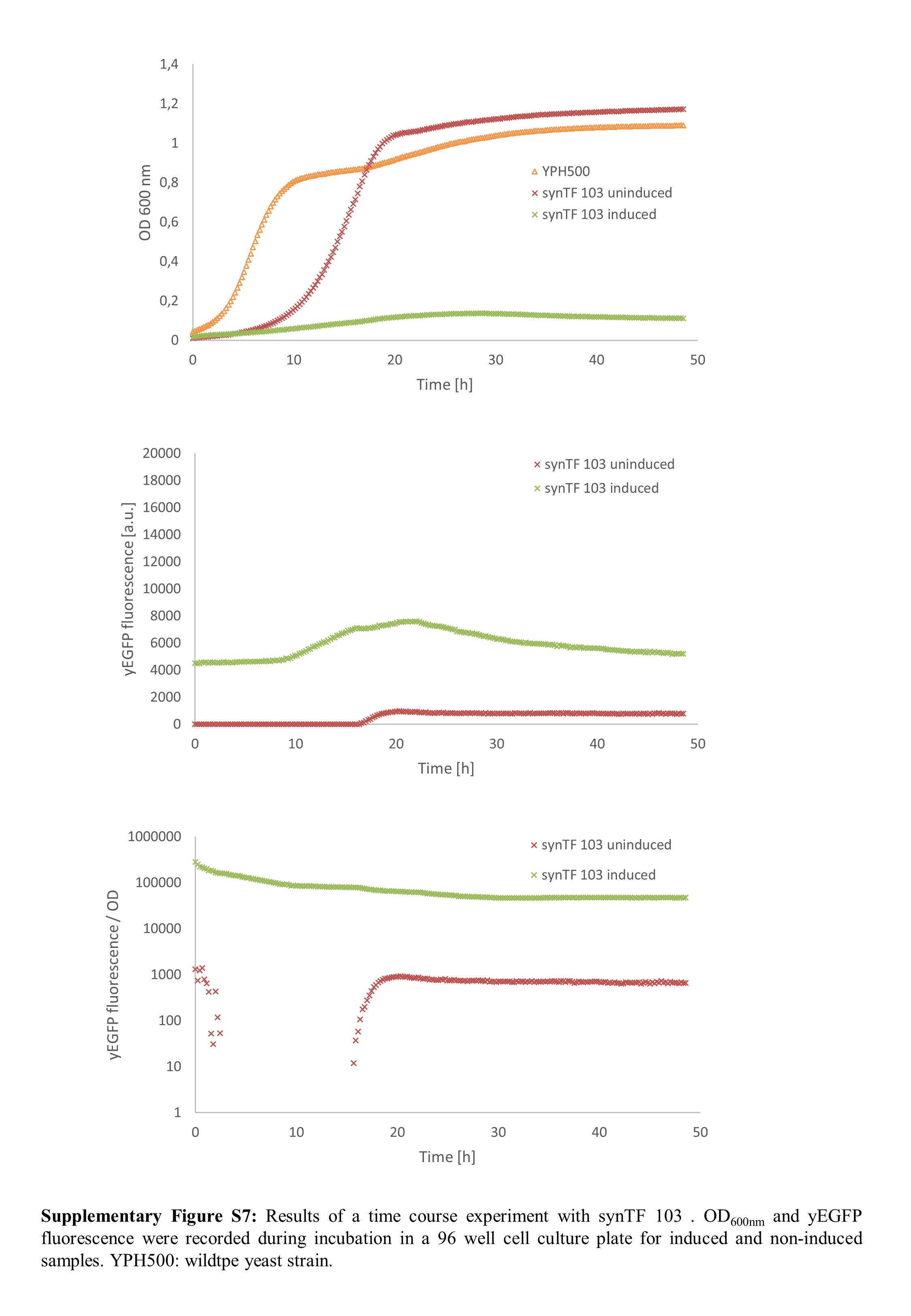

Supplement: Supplementary file 13 [file Image_7.JPEG]

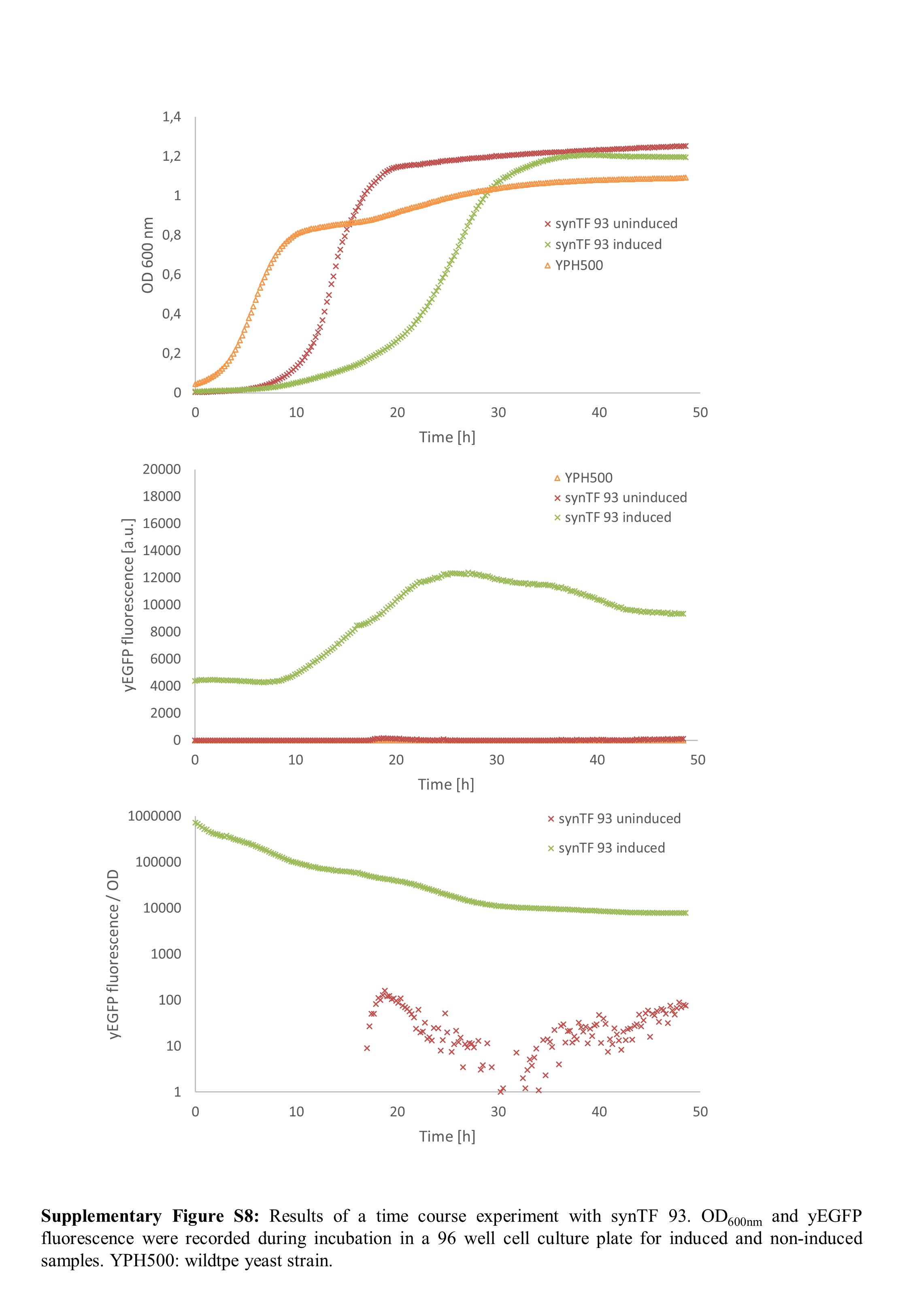

Supplement: Supplementary file 14 [file Image_8.JPEG]

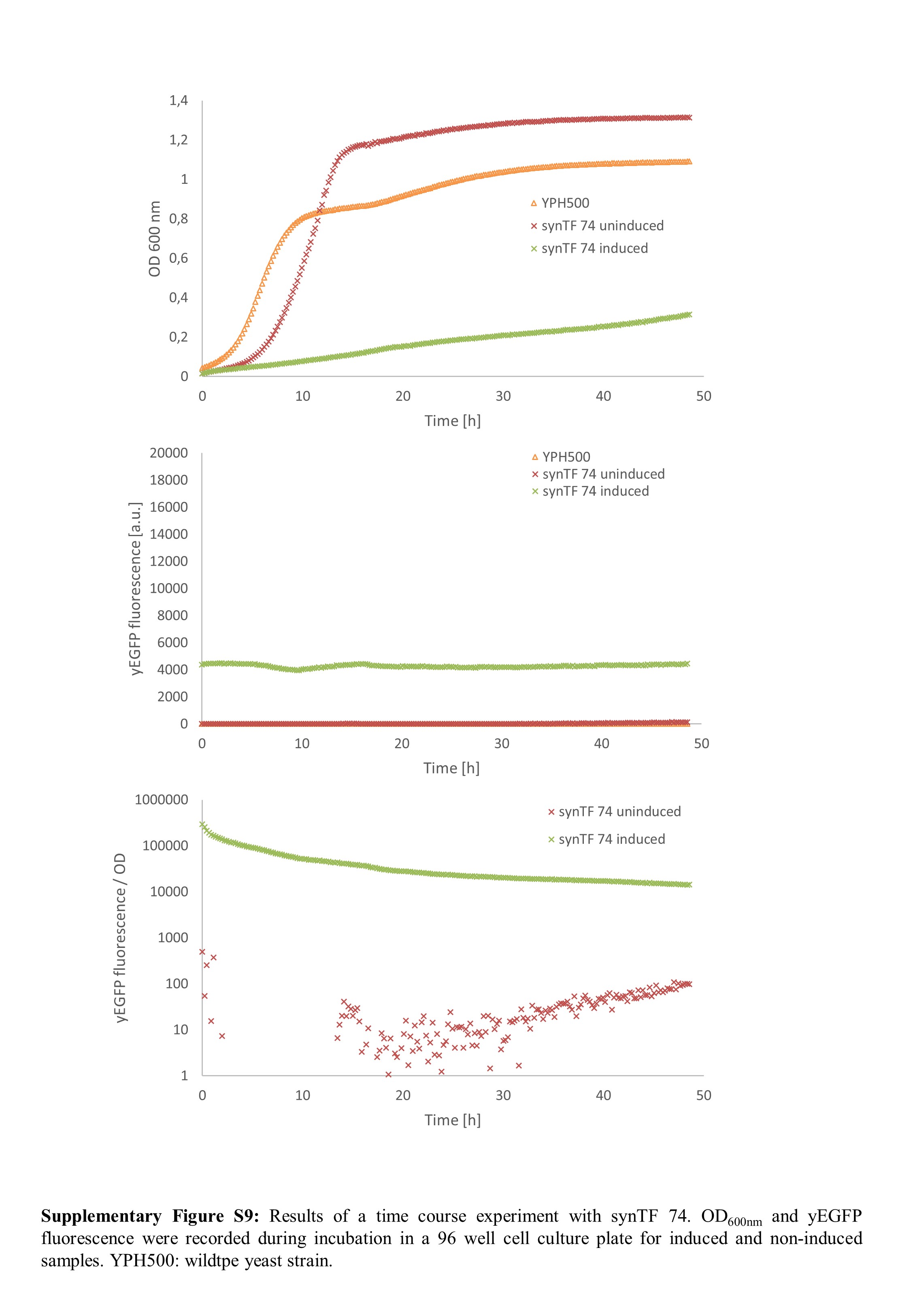

Supplement: Supplementary file 15 [file Image_9.JPEG]

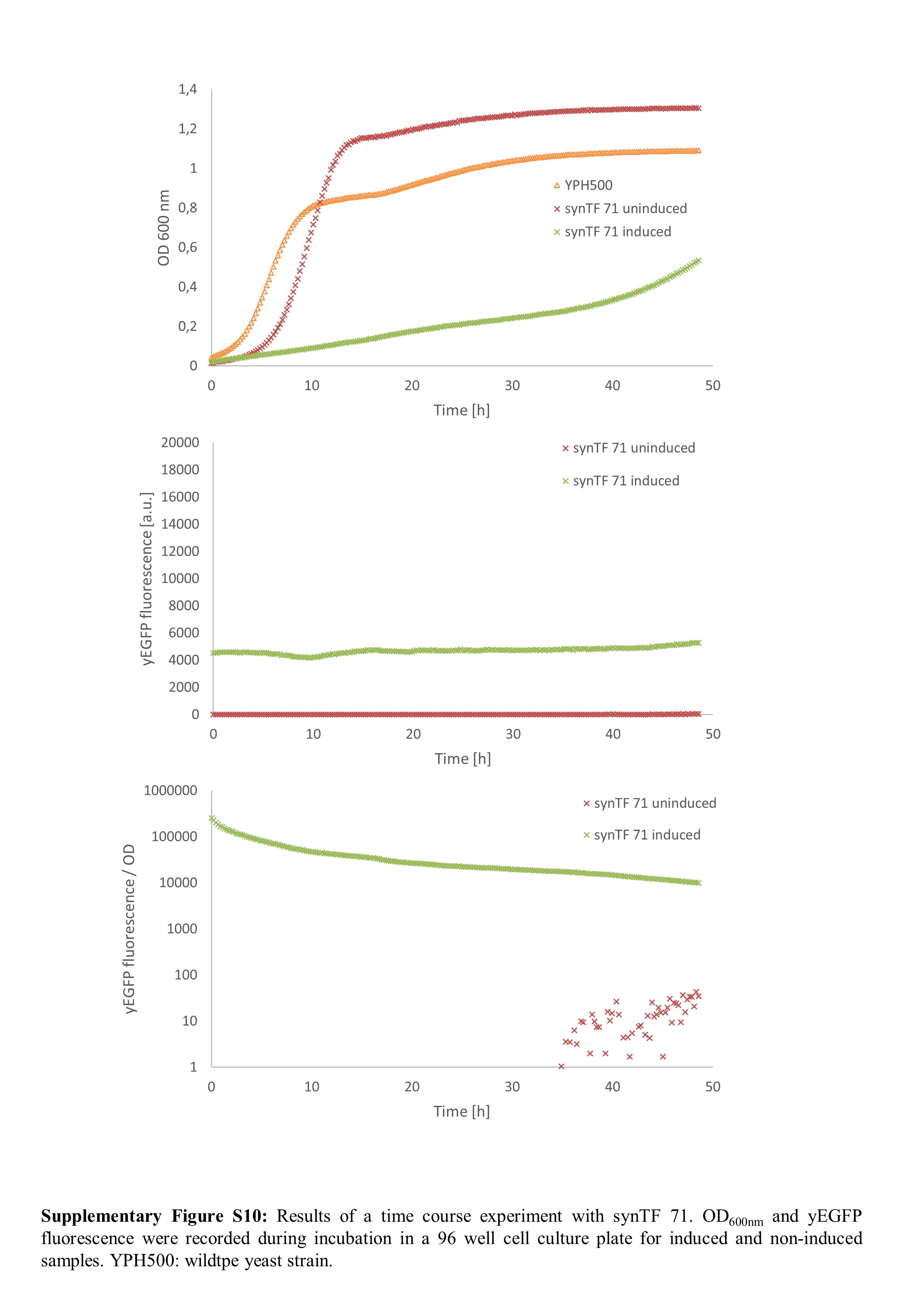

Supplement: Supplementary file 16 [file Image_10.JPEG]

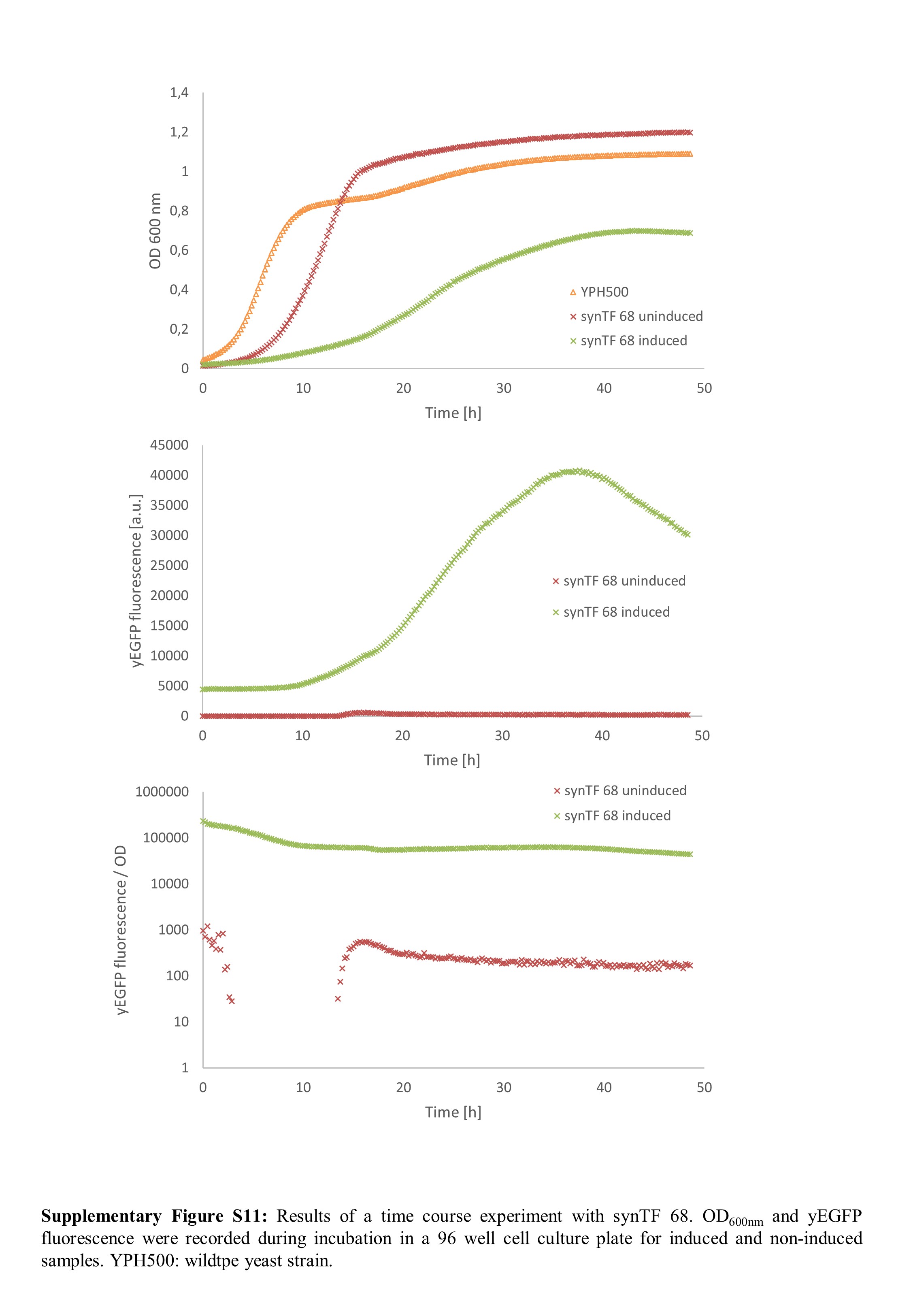

Supplement: Supplementary file 17 [file Image_11.JPEG]

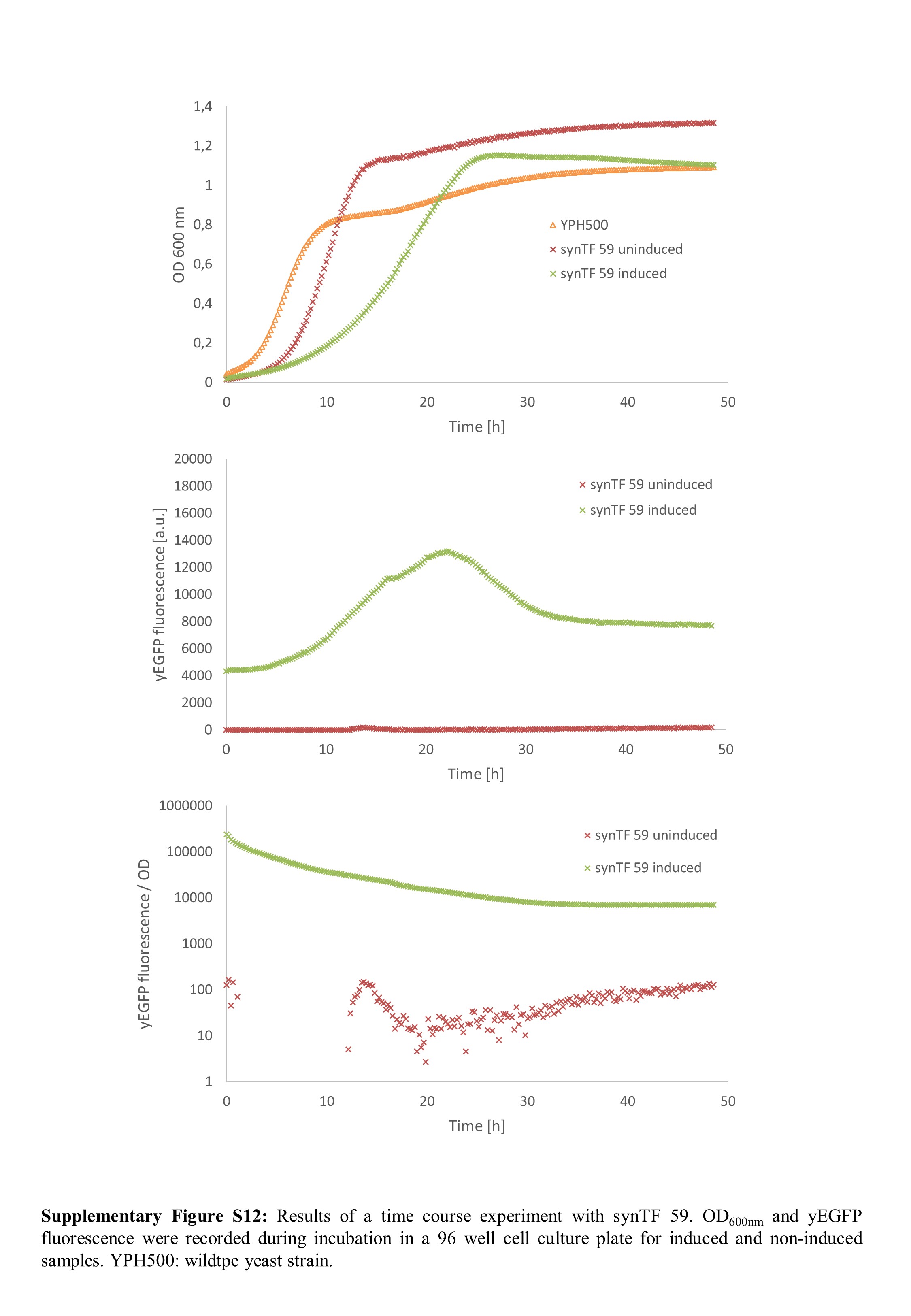

Supplement: Supplementary file 18 [file Image_12.JPEG]

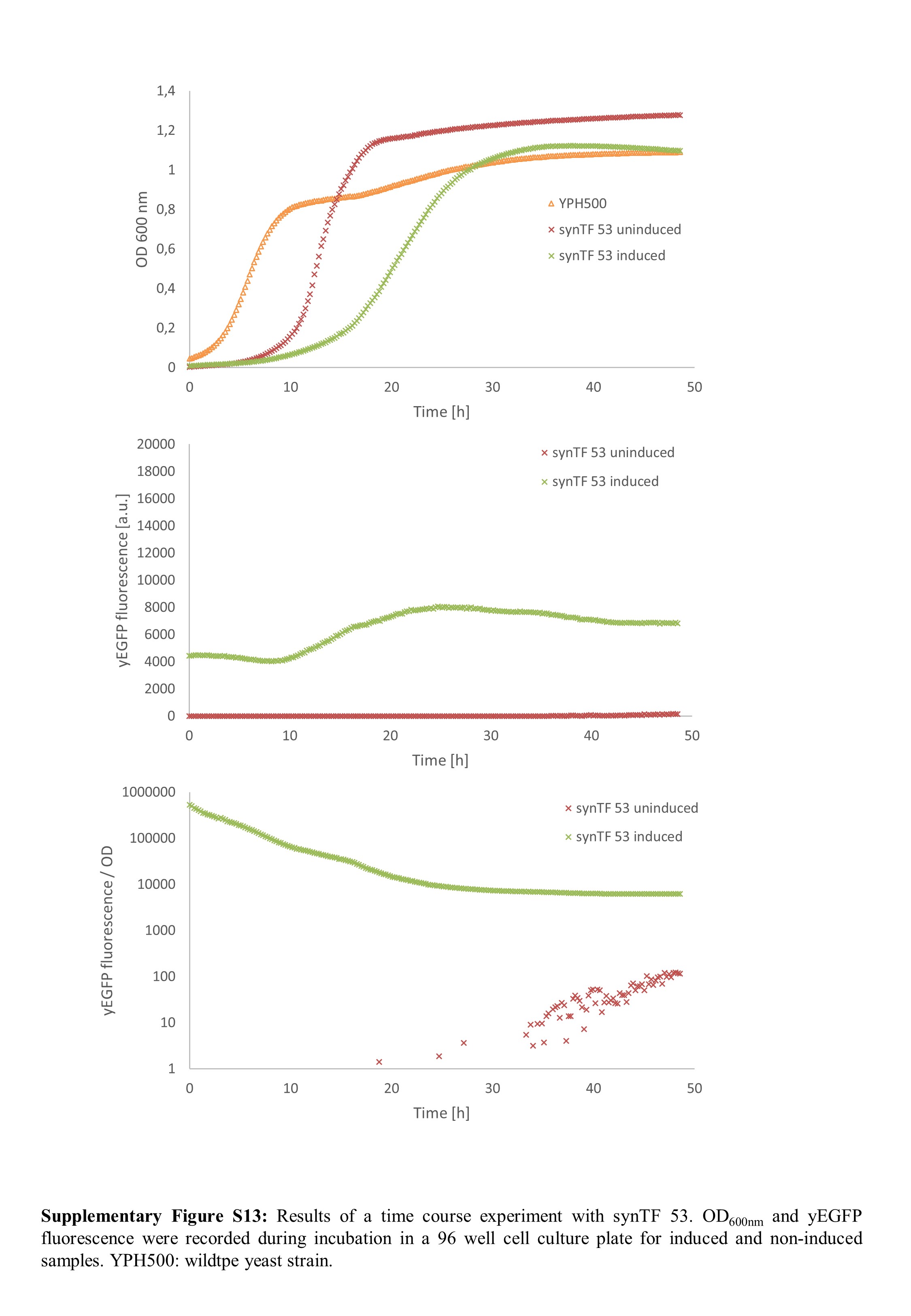

Supplement: Supplementary file 19 [file Image_13.JPEG]

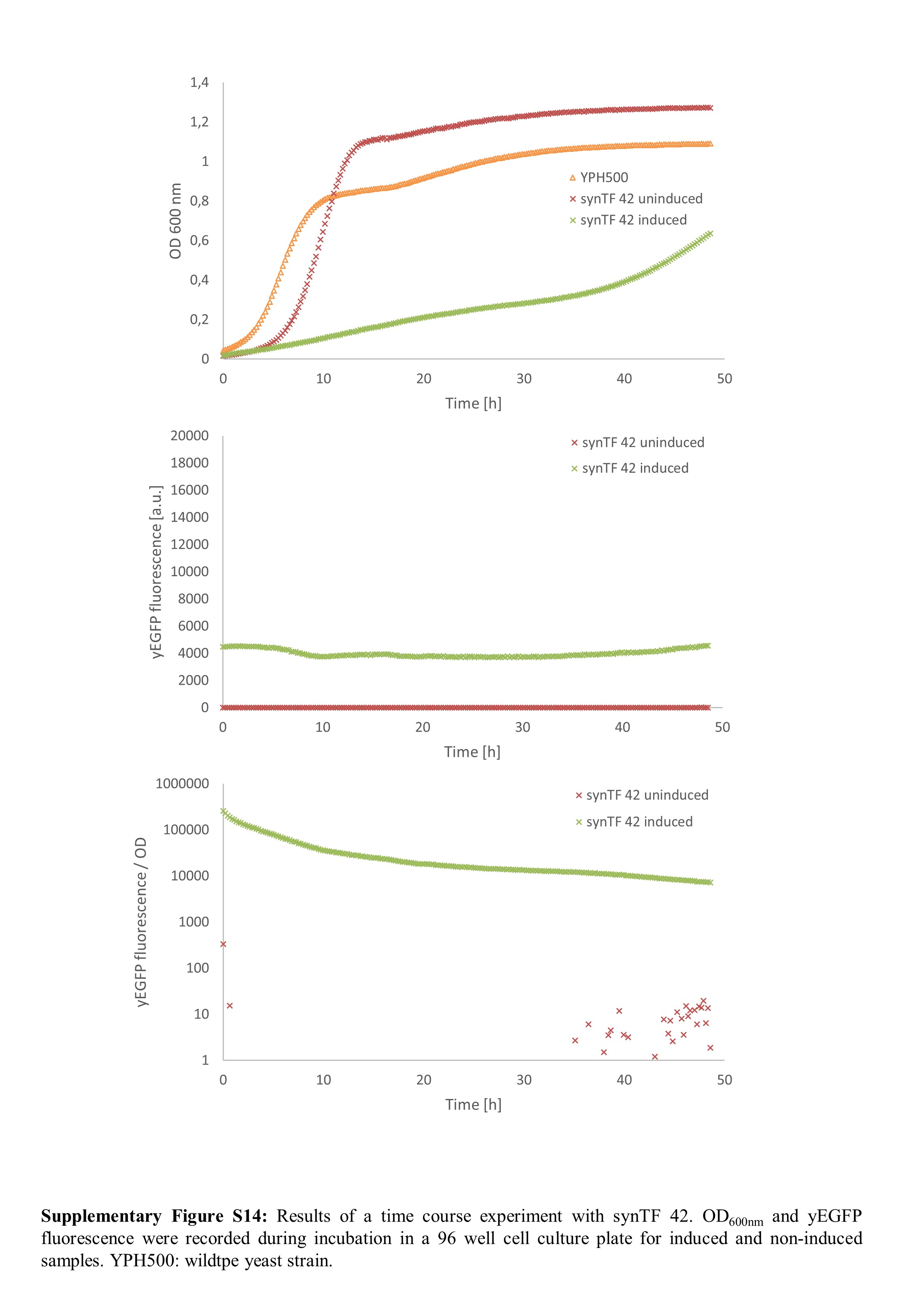

Supplement: Supplementary file 20 [file Image_14.JPEG]

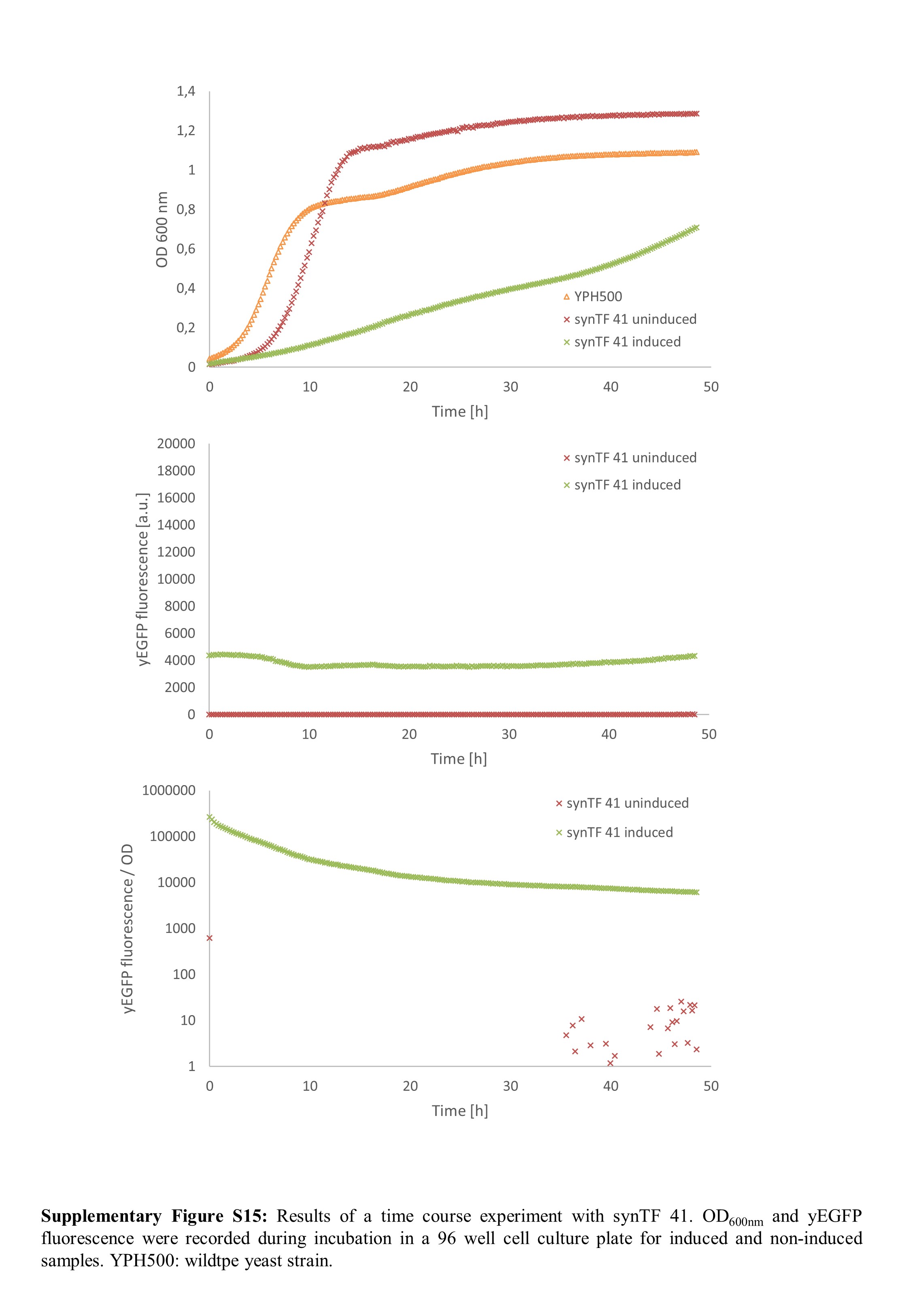

Supplement: Supplementary file 21 [file Image_15.JPEG]
